# Lactobacillus rhamnosus GG ameliorates hyperuricemia in a novel model

**DOI:** 10.1038/s41522-024-00486-9

**Published:** 2024-03-20

**Authors:** Yang Fu, Yong-Song Chen, Dai-Yang Xia, Xiao-Dan Luo, Hao-Tong Luo, Jie Pan, Wei-Qing Ma, Jin-Ze Li, Qian-Yuan Mo, Qiang Tu, Meng-Meng Li, Yue Zhao, Yu Li, Yi-Teng Huang, Zhi-Xian Chen, Zhen-Jun Li, Lukuyu Bernard, Michel Dione, You-Ming Zhang, Kai Miao, Jian-Ying Chen, Shan-Shan Zhu, Jie Ren, Ling-Juan Zhou, Xian-Zhi Jiang, Juan Chen, Zhen-Ping Lin, Jun-Peng Chen, Hui Ye, Qing-Yun Cao, Yong-Wen Zhu, Lin Yang, Xue Wang, Wen-Ce Wang

**Affiliations:** 1 State Key Laboratory of Swine and Poultry Breeding Industry, College of Animal Science, South China Agricultural University, Guangzhou, 510642 China; 2 Clinical Research Center, The First Affiliated Hospital of Shantou University Medical College, Shantou, 515041 China; 3 School of Marine Sciences, Sun Yat-sen University, and Southern Marine Science and Engineering Guangdong Laboratory (Zhuhai), Zhuhai, 519082 China; 4 Hunan Shihua Biotech Co. Ltd., Changsha, 410000 China; 5 State Key Laboratory of Microbial Technology, Shandong University, Qingdao, 266237 Shandong China; 6 School of Agricultural Science and Engineering, Liaocheng University, Liaocheng, 252000 China; 7 Key Laboratory of Carcinogenesis and Translational Research, Departments of Lymphoma, Radiology and Nuclear Medicine, Peking University Cancer Hospital and Institute, Beijing, 100080 China; 8 International Livestock Research Institute, Nairobi, 00100 Kenya; 9 Cancer Center, Faculty of Health Sciences, University of Macau, Macau, SAR China; 10 Department of Rheumatology and Immunology, The First Affiliated Hospital of Jinan University, Guangzhou, 510630 China; 11 Microbiome Research Center, Moon (Guangzhou) Biotech Co. Ltd., Guangzhou, 510535 China; 12 Shantou Baisha Research Institute of Origin Species of Poultry and Stock, Shantou, 515041 China

**Keywords:** Next-generation sequencing, Metagenomics, Microbiota

## Abstract

Hyperuricemia (HUA) is a metabolic syndrome caused by abnormal purine metabolism. Although recent studies have noted a relationship between the gut microbiota and gout, whether the microbiota could ameliorate HUA-associated systemic purine metabolism remains unclear. In this study, we constructed a novel model of HUA in geese and investigated the mechanism by which *Lactobacillus rhamnosus* GG (LGG) could have beneficial effects on HUA. The administration of antibiotics and fecal microbiota transplantation (FMT) experiments were used in this HUA goose model. The effects of LGG and its metabolites on HUA were evaluated in vivo and in vitro. Heterogeneous expression and gene knockout of LGG revealed the mechanism of LGG. Multi-omics analysis revealed that the *Lactobacillus* genus is associated with changes in purine metabolism in HUA. This study showed that LGG and its metabolites could alleviate HUA through the gut-liver-kidney axis. Whole-genome analysis, heterogeneous expression, and gene knockout of LGG enzymes ABC-type multidrug transport system (*ABCT*), inosine-uridine nucleoside N-ribohydrolase (*iunH*), and xanthine permease (*pbuX*) demonstrated the function of nucleoside degradation in LGG. Multi-omics and a correlation analysis in HUA patients and this goose model revealed that a serum proline deficiency, as well as changes in *Collinsella* and *Lactobacillus*, may be associated with the occurrence of HUA. Our findings demonstrated the potential of a goose model of diet-induced HUA, and LGG and proline could be promising therapies for HUA.

## Introduction

Hyperuricemia (HUA) has become a worldwide metabolic disease with increased prevalence in most countries^1^. The prevalence of HUA is particularly high in coastal and oceanic areas, such as the USA (20%), Japan (25%), and European countries (19–25%)^2^. According to the Chinese guidelines for the diagnosis and treatment of HUA and gout, the number of HUA patients in China increased by 30% between 1998 and 2018, and the number of gout patients increased by 2.18-fold. (Supplementary Fig. 1b). With an elevation of the serum uric acid (UA) concentration of more than 420 μmol/L (7 mg/dL), HUA is the primary risk factor for the development of gout, chronic kidney disease, type 2 diabetes, and cardiovascular disease^3–5^. Global Burden of Disease data show that gout-related YLDs (Years Lived with Disability) have increased steadily from 1990 to 2019 (Supplementary Fig. 1a). UA is a purine nucleoside degradation end-product, generated from exogenous or endogenous nucleic acid, nucleotide, and purine-containing compounds. The serum UA level is greatly influenced by dietary factors, such as a high-purine diet, fructose, and alcohol intake, accompanied by an imbalance of UA generation and excretion^6^.

Since they lack uricase, both humans and poultry are unable to degrade UA and are vulnerable to HUA or gout^7,8^. However, in most mammals, including mice, the risk of developing HUA is negligible due to the presence of uricase. Mouse models have been widely used in studies on HUA or gout, but with several challenges, including the difficulty of defining HUA in a setting of variable UA levels, the difficulty of modeling through uricase inhibition or direct purine injection rather than through changes in diet, and the key point that there are differences in purine metabolism and urate excretion^9^. The establishment of reliable and consistent diet-induced HUA animal models has been a long-standing goal for researchers. Among poultry, geese are highly susceptible to gout, and gout in goslings carries high morbidity and mortality rates^10^. Around 500–600 million geese are consumed in China every year and more than 50% of these geese will suffer gout during the growing period^11,12^. The mechanism of causation, pathogenesis, and symptoms of HUA in geese are identical to those in humans. Excessive protein (high-purine) consumption in the diet is strongly associated with an increased risk of diet-derived HUA and gout in these two species^13,14^.

**Fig. 1:**
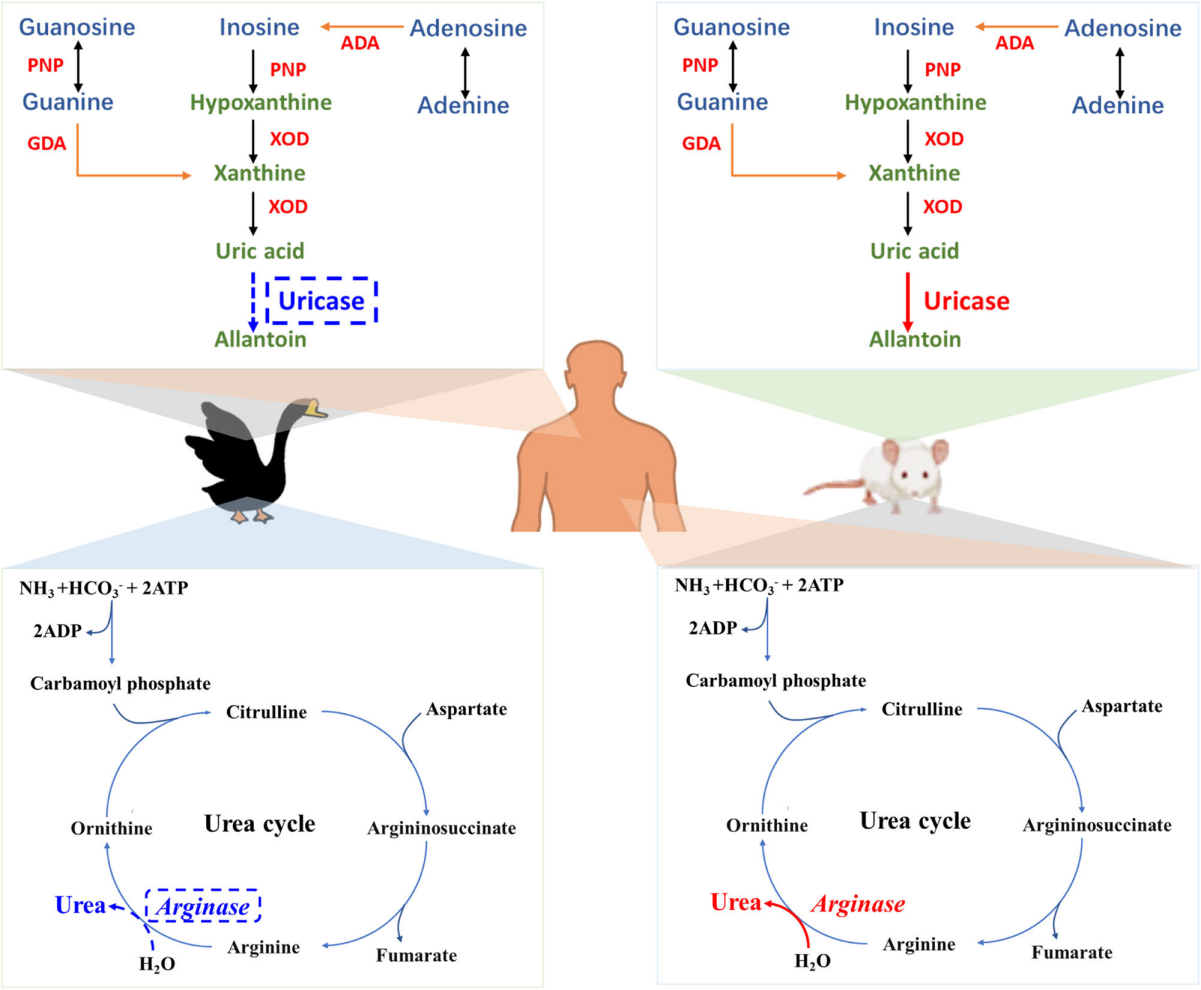
Goose is a more ideal HUA model because of its specific physiological defects. Compared with mice, geese and humans have one innate commonality: a lack of uricase, which is especially important for the formation of hyperuricemia. Fortunately, people can excrete a part of the ammonia produced by protein degradation through the intestinal-hepatic urea cycle, thus reducing the production of a piece of uric acid. In contrast, geese are more susceptible to HUA because their liver lacks arginase and cannot synthesize urea. In summary, the goose is an ideal HUA model.

Strategies to modulate the gut microbiota distribution have been intensively studied for diseases associated with changes to the gut microbiota (dysbiosis), such as diarrhea, HUA, obesity, and metabolic syndrome^15–17^. Emerging evidence demonstrates that gut microbiota dysbiosis is associated with abnormal urate degradation and systemic inflammation in HUA and gout patients. Clinic parameters and metagenomic analyses have revealed that members of *Enterobacteriaceae* contribute to the reduction of UA in healthy individuals^18^. *Bacteroides caccae* and *Bacteroides xylanisolvens* are abundant in patients^19^. The gut microbiota in HUA plays a role in nucleotide and amino acid metabolism, which makes intestinal microbes a potential target for treating HUA and gout^18,20^. Recent research has demonstrated that some gut microbes participate in the mechanism of UA degradation. For example, the oral administration of *Limosilactobacillus fermentum* and *Lactiplantibacillus plantarum* isolated from fermented food has been shown to strongly degrade UA in mice^21,22^. *Lactobacillus gasseri* PA3 can decrease the UA concentration and increase the relative abundance of *Lactobacillus*^23,24^. *Lactobacillus rhamnosus* GG (LGG), a gastrointestinal probiotic strain^25^, has been reported to have beneficial effects with regard to metabolic diseases such as diabetes^26^, hypertension^27^, insulin resistance^28^, and obesity^29^. Studies have shown that LGG ameliorates metabolic disorders in the body mainly by affecting in the intestinal microbiota^30^, such as decreasing opportunistic bacteria (*Bacteroidetes* and *Proteobacteria*) and increasing beneficial bacteria (*Lactobacillus*, *Bifidobacterium*, *Butyricicoccus*)^31–33^. However, the specific mechanism by which LGG alleviates HUA has not been elucidated. Although it has been reported that species from different sources contribute to UA degradation in the gut, the mechanism of the degradation of UA by bacteria in the gut is not fully elucidated and the effect on gut metabolites remains undefined (Fig. 1).

Here, we established a novel model of diet-borne HUA in geese and treated the animals with antibiotics and FMT to shed light on the role of the gut microbiota in diet-borne HUA. Dietary supplementation and oral gavage experiments revealed that LGG had mitigating effects in the foodborne-induced HUA goose model through the gut-liver-kidney axis. Three key genes associated with nucleoside and purine degradation in LGG, *ABCT*, *iunH*, and *pbuX*, were identified by genome sequencing and analysis. Heterogeneous expression and knockdown of these genes demonstrated the mechanism of UA degradation by LGG. Both LGG metabolites and proline alleviated intestinal, hepatic, and renal dysfunction in vitro. A metagenome analysis in gout patients and healthy controls showed purine metabolism features similar to those in the HUA goose model. Collectively, our results strengthen the association between gut microbes and HUA and provide a possible probiotic therapy for HUA through the gut microbiota.

## Results

### HCP diet disturbed the gut microbiota and induced HUA in goslings through the gut-liver-kidney axis

**Fig. 2:**
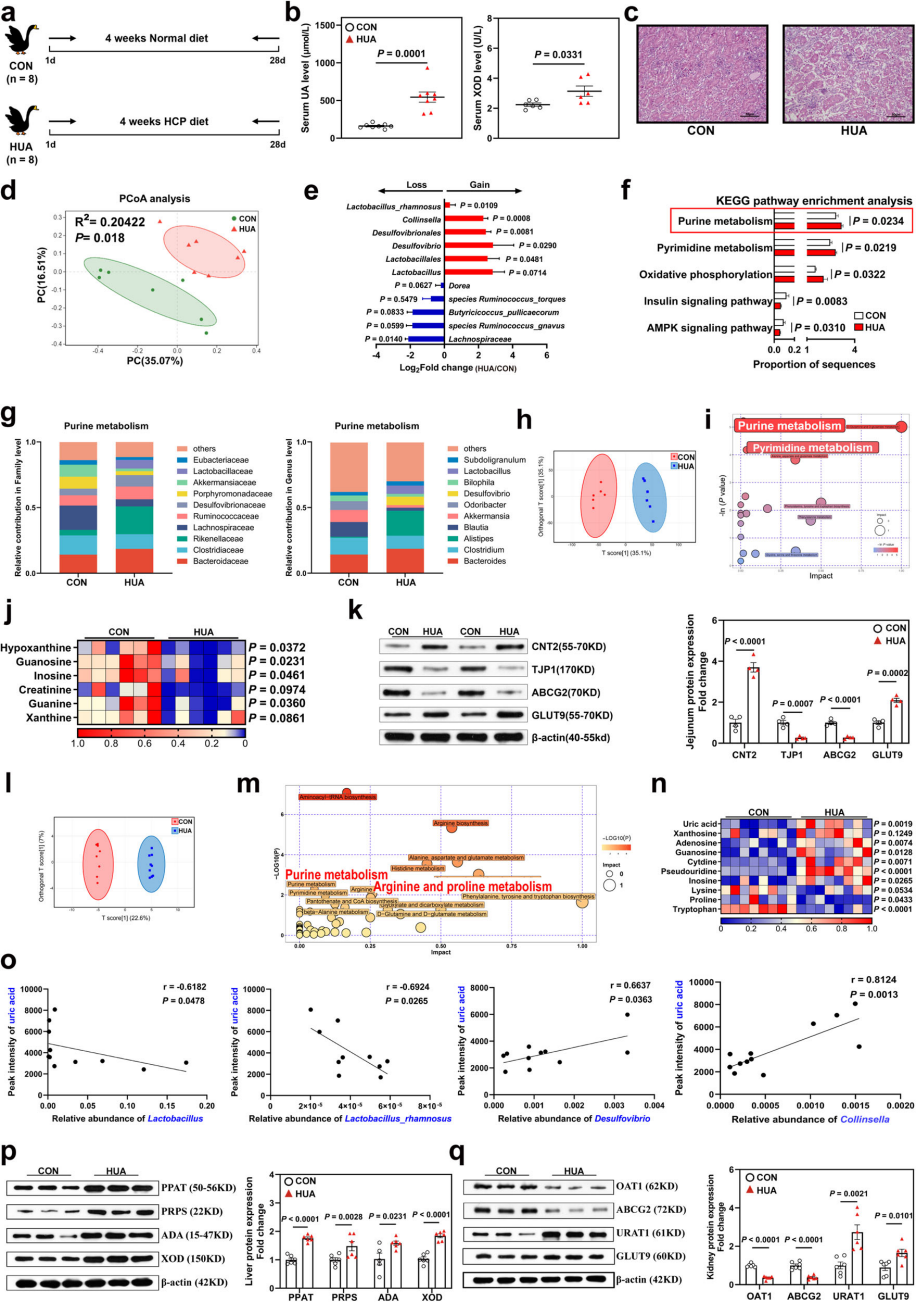
HCP diet disturbs gut flora and constructs a gosling HUA model through the gut-liver-kidney axis. **a** Experimental design. One-day-old goslings were selected and divided into two groups to be fed a normal diet and HCP diet respectively for 28 d. **b** Effect of HCP diet on the serum uric acid (UA) (*n* = 8, mean with SEM), xanthine oxidase (XOD) levels (*n* = 6, mean with SEM). **c** Representative image of H&E staining of kidney sections from CON group or HUA group (×400, *n* = 8). The white part of the peripheral renal tubule in the right figure is the proteinuria protein cast lesions. All scale bars are 50 μm. **d** Principal components analysis of bacteria with 95% confidence regions between CON group (*n* = 7, green) and HUA group (*n* = 7, red). **e** The alteration trends of the bacterial relative abundance (*n* = 7). The x-axis shows the log2 fold change of the bacterial relative abundance in the HUA group compared to the CON group. **f** The abundance of microbial function genes and gene families in the CON group (white) and HUA group (red) (*n* = 7, mean with SEM). **g** Changes in the functional contribution of purine metabolism (top ten bacterial in terms of abundance, left: Family level, right: Genu level). **h** OPLS-DA of the fecal samples (*n* = 6). The red color represents the CON group, while the blue color HUA group. Compounds that were selected through RP and HILIC were analyzed separately. **i** KEGG pathway enrichment differential fecal metabolites between CON group and HUA group (*n* = 6). The y-axis shows the -Ln *P*-value, two significant pathways with *P* < 0.05 were highlighted with their names. **j**, Heatmap of LC-MS data showing fecal purine metabolite changes under HCP diet (*n* = 6). Increases in metabolite levels are shown in red, whereas blue indicates decreased metabolite. **k** Representative western blotting images and quantification of proteins (CNT2, TJP1, ABCG2, GLUT9) in the jejunum tissue between the CON group and HUA group (*n* = 4). **l** OPLS-DA of the serum samples (*n* = 8). The red color represents the CON group, while the blue color HUA group. Compounds that were selected through RP and HILIC were analyzed separately. **m** KEGG pathway enrichment differential serum metabolites between CON group and HUA group (*n* = 8). The y-axis shows the -Ln *P*-value, two significant pathways with *P* < 0.05 were highlighted with their names. **n** Heatmap of LC-MS data showing serum purine and amino acid metabolite changes under HCP diet (*n* = 8). Increases in metabolite levels are shown in red, whereas blue indicates decreased metabolite. **o** Pearson correlation analysis between gut microbiome relative abundance and serum metabolite relative level. **p** Representative western blotting images and quantification of proteins (PPAT, PRPS, ADA, XOD) in the liver tissue between the CON group and HUA group (*n* = 6). **q** Representative western blotting images and quantification of proteins (OAT1, ABCG2, URAT, GLUT9) in the kidney tissue between the CON group and HUA group (*n* = 6, mean with SEM). Data with error bars represent mean ± s.e.m. For (**b**, **e**, **j**, **k**) and (**o**, **p**, **q**), data were analyzed by two-tailed unpaired Student’s *t*-test. For (**f**), data were employed for the Wilcoxon rank-sum test.

We established an HUA model by feeding 1-day-old male goslings with a high calcium and protein (HCP) diet (Fig. 2a and Table 1). A 3.2-fold increase in UA levels was detected in the HUA group compared with the control (CON) group (*P* = 0.0001, Fig. 2b), which greatly exceeded the threshold of HUA in geese. The serum creatinine (Cr, *P* = 0.0398), xanthine oxidase (XOD, *P* = 0.0331), and calcium (Ca, *P* = 0.0257) levels were all up-regulated and the phosphorus (P, *P* = 0.0077) level was down-regulated (Fig. 2b, Supplementary Fig. 2a). Nephrotic morphology showed that protein casts dilated tubules in the HUA kidney, which are the typical renal lesions seen in HUA and gout (Fig. 2c). These characteristics suggest that pathological symptoms typical of HUA are seen in geese induced by the HCP diet.

**Table 1:** Composition and nutrient content of standard diet in each group (air-dry basis, %)

Ingredient (%)	CON^d^	HUA^c^	Nutrient level (%)^b^	CON	HUA
Corn	45.95	40.0	ME (MJ/kg)	12.15	12.14
Corn protein powder	7.00	13.33	CP^e^ (%)	16.81	24.03
Fish meal	0.00	6.20	CF^f^ (%)	3.96	3.55
Soybean meal	13.79	18.00	Ca^g^ (%)	1.00	3.04
Wheat bran	15.00	0.00	Non-phytate P (%)	0.50	0.64
Rice bran	7.20	9.80	Threonine (%)	0.76	0.93
Rice husk	2.00	2.20	Methionine (%)	0.57	0.56
Fat powder	1.00	1.00	Met + Cys (%)	0.85	0.90
NaCl	0.30	0.30	Tryptophan (%)	0.19	0.27
CaHPO_4_	1.6	1.62			
Limestone	1.50	6.25			
DL-Methionine (98%)	0.30	0.10			
L-Threonine	0.20	0.10			
L-Lys-HCL (78%)	0.20	0.10			
Premix^a^	1.00	1.00			
Bentonite	2.96	0			
Total	100	100			

^a^Premix supplied the following constituents per kilogram of complete diet: VA 12000 IU, VD_3_ 33000 IU, Niacinamide 60 mg, VE 30 mg, D-Pantothenic acid 18 mg, VB_2_ 9 mg, VK 6 mg, VB_6_ 6 mg, VB_12_ 0.03 mg, VB_1_ 3 mg, Folic acid 1.5 mg, Biotin 0.15 mg, Ethoxyquin 0.5 mg, Cu (CuSO_4_·5H_2_O) 8 mg, Fe (FeSO_4_·7H_2_O) 80 mg, Zn (ZnSO_4_·7H_2_O) 90 mg, Mn (MnSO_4_·H_2_O) 70 mg, Co (CoSO_4_·7H_2_O) 0.32 mg, Se (NaSeO_3_) 0.32 mg, I (KI) 0.45 mg.

^b^Crude protein and calcium are measured values, the rest of the nutrient levels are calculated values.

^c^High calcium and high protein diets.

^d^Control diets.

^e^CP: crude protein.

^f^CF: crude fiber.

^g^Ca: calcium.

To study the changes in the gut microbiota in the HUA goose model, we performed a metagenomics analysis in the CON and HUA groups. The gut microbiota composition in the HUA group was quite different from that in the CON group. A principal coordinate coordinates analysis (PCoA) uncovered a significant difference in the gut microbiota between the HUA and CON groups (*R*^2^ = 0.2042, *P* = 0.0180; Fig. 2d). The total abundance of bacteria in the CON group was greater than that in the HUA group (Supplementary Fig. 2b). The HCP diet reduced the relative abundance of *Lachnospiraceae* (*P* = 0.0140), *Butyricicoccus pullicaecorum* (*P* = 0.0833), *Ruminococcus torques* (*P* = 0.5479), *Ruminococcus gnavus* (*P* = 0.0599), and *Dorea* (*P* = 0.0627), and increased the relative abundance of *Collinsella* (*P* = 0.0008) and *Desulfovibrionales* (*P* = 0.0081). Notably, the relative abundances of *Lactobacillus* (*P* = 0.0714) and LGG (*P* = 0.0109) in the HUA group were increased (Fig. 2e). The linear discriminant analysis effect size (LEfSe) based on the Kyoto Encyclopedia of Genes and Genomes (KEGG) database showed that the purine metabolism function of intestinal microbiota was significantly up-regulated (Wilcoxon rank sum test, *P* = 0.0234; Fig. 2f and Supplementary Fig. 2c). Furthermore, the results regarding species contribution based on purine metabolism function revealed that the families *Bacteroidaceae*, *Rikenellaceae*, *Desulfovibrionaceae*, and *Lactobacillaceae*, as well as the genera *Bacteroides*, *Alistipes*, *Desulfovibrio* and *Lactobacillus* significantly contributed to purine metabolism in the HUA group (Fig. 2g).

We also performed a metabolomic analysis of cecal chyme to demonstrate the purine metabolism in the intestine. An orthogonal partial least squares discriminant analysis (OPLS-DA) revealed a distinct difference in cecal chyme metabolites between the HUA and CON groups (Fig. 2h). A pathway enrichment analysis demonstrated that these metabolites were primarily associated with purine metabolism and pyrimidine metabolism, with purine metabolism emerging as the pivotal hub (Fig. 2i). However, the abundance of UA precursor metabolites, such as xanthine, Hypoxanthine (HX), guanine, guanosine, and inosine, in cecal chyme was significantly reduced (Fig. 2j). Microbial purine metabolism was enhanced, but the related metabolites in cecal chyme were decreased. Expression of the intestinal UA reabsorption transporter glucose transporter 9 (GLUT9), and concentrative nucleoside transporter type 2 (CNT2) was up-regulated in the HUA group, while expression of the UA excretion transporter ATP-binding cassette transporter G2 (ABCG2) and intestinal tight junction protein 1 (TJP1) was down-regulated (Fig. 2k). Consistent with these results, Hematoxylin-eosin (HE) staining showed impairment of the intestinal morphology (Supplementary Fig. 2d, e). These results demonstrated that further weakening of the intestinal barrier accelerates the translocation of intestinal pathogens and UA metabolites.

To further investigate the impact of HUA on serum, we performed an untargeted metabolomic analysis of serum metabolites in the HUA and CON groups. OPLS-DA showed that the two groups had distinct metabolite profiles (Fig. 2l). A pathway enrichment analysis demonstrated that the identified metabolites were primarily associated with purine metabolism and arginine/proline metabolism (Fig. 2m). The HCP diet significantly increased levels of UA and UA precursor metabolites, such as adenosine, guanosine, and inosine in serum (Fig. 2n). These results further demonstrated the translocation of UA precursor metabolites from the intestine to serum. Meanwhile, HUA also disrupted amino acid metabolism, significantly decreasing levels of proline and tryptophan (Fig. 2n, Supplementary Fig. 2f). Additionally, a Pearson correlation analysis indicated that the genus *Lactobacillus* (*P* = 0.0478) and species LGG (*P* = 0.0265) had a substantially negative association with the serum UA level, while the genera *Desulfovibrio* (*P* = 0.0363) and *Collinsella* (*P* = 0.0013) had a significantly positive correlation with UA (Fig. 2o). Although the relative abundance of the genus *Lactobacillus* and species LGG were increased in the HUA group, these adverse results demonstrated that they may reduce the serum UA level, while the genera *Desulfovibrio* and *Collinsella* are implicated in an increase in UA.

We further investigated UA synthesis-related genes in the liver. HUA significantly up-regulated the protein expression of UA synthesis-related enzymes PRPS, PPAT, ADA, and XOD (Fig. 2p). Meanwhile, liver inflammatory infiltration in the HUA group was observed by HE staining, and the number of inflammatory cells was significantly increased (Supplementary Fig. 2h). The main catalytic function of XOD is the conversion of HX to xanthine and subsequently to UA. Moreover, liver IL-1β, IFN-γ, and TNF-α levels in the HUA group were much higher than those in the CON group. There was a substantial increase in serum IL-1β and IFN-γ, indicating the presence of systemic inflammation in HUA geese (Supplementary Fig. 2i).

In additional to the nephritic tubulointerstitial lesions described above, we also found a significant down-regulation of UA excretion proteins OAT1 and ABCG2 and the increased expression of UA resorption protein URAT1 and GLUT9 in the HUA group (Fig. 2q).

Taken together, these results suggest that the HCP diet-induced HUA in goslings led to dysbiosis of the gut, microbiota and disrupted purine metabolism, along with UA synthesis in the gut and liver. HUA also induced the inhibition of UA excretion in the kidney.

### Antibiotic treatment altered the gut microbiota and ameliorated HCP diet-induced HUA through the gut-liver-kidney axis

**Fig. 3:**
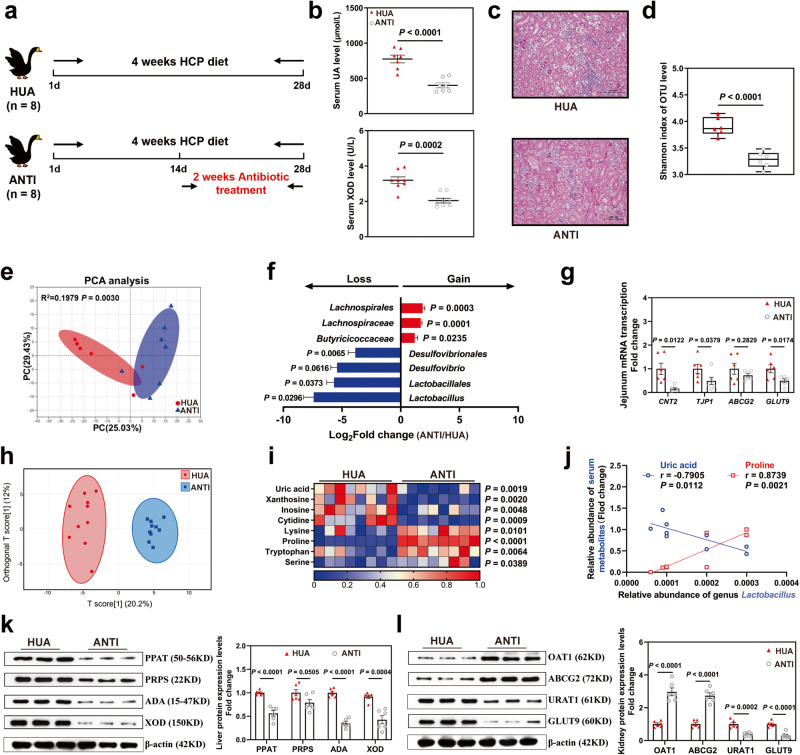
Antibiotic treatment adjusts gut flora and alleviates HCP diet-induced HUA through the gut-liver-kidney axis. **a** Experimental design. One-day-old goslings were selected and divided into two groups to be fed HCP diet for 4 weeks and then ANTI group gavage of antibiotics for 2 weeks. **b** Effect of antibiotics on the serum UA, and XOD levels in HCP diet-treated geese (*n* = 8). **c** Representative image of H&E staining of kidney sections from HUA group and ANTI group (×400, *n* = 8). The white part of the peripheral renal tubule in the right figure is the proteinuria protein cast lesions. All scale bars are 50 μm. **d** Shannon index of indicated groups based on alpha diversity analysis (*n* = 6). **e** Principal components analysis of bacteria with 95% confidence regions between the HUA group (red, *n* = 6) and ANTI group (blue, *n* = 7). **f** The alteration trends of the bacterial relative abundance (*n* = 7). The x-axis shows the log2 fold change of the bacterial relative abundance in the ANTI group compared to the HUA group. **g** Effect of antibiotics treatment on the relative expression of nucleoside transport gene (CNT2), gut barrier gene (TJP1), UA excretion genes (ABCG2), and reabsorption genes (GLUT9) in jejunum tissue by RT-PCR analysis (*n* = 6). **h** OPLS-DA of the serum samples (*n* = 10). The red color represents the HUA group, while the blue color ANTI group. Compounds that were selected through RP and HILIC were analyzed separately. **i** Heatmap of LC-MS data showing serum purine and amino acid metabolite changes between the HUA group and ANTI group (*n* = 8). Increases in metabolite levels are shown in red, whereas blue indicates decreased metabolite. **j** Pearson correlation analysis between *Lactobacillus* relative abundance and serum metabolite relative level. **k** Representative western blotting images and quantification of proteins (PPAT, PRPS, ADA, XOD) in the liver tissue of HCP diet-treated geese (*n* = 6) between the HUA group and ANTI group. **l** Representative western blotting images and quantification of proteins (OAT1, ABCG2, URAT, GLUT9) in the kidney tissue between the HUA group and ANTI group (*n* = 6). Data with error bars represent mean ± s.e.m. For (**b**, **d**, **g**, **i**, **k**, and **l**), data were analyzed by two-tailed unpaired Student’s *t*-test.

To further investigate the association between HUA and the microbiota, we designed a pseudo-sterile animal experiment in which antibiotics were administered (Fig. 3a). Intriguingly, we observed a reversal of HUA symptoms following antibiotic treatment, characterized by a significant reduction of BUN, Cr, XOD, and UA accumulation in serum (Fig. 3b, Supplementary Fig. 3a), as well as the alleviation of renal tubular necrosis (Fig. 3c).

Subsequently, we performed a 16S rRNA gene sequence analysis of cecum chyme and found that the antibiotic group had a lower Shannon index (*P* < 0.0001, Fig. 3d), and a Principal Component Analysis revealed a clear difference between the HUA and ANTI groups (*R*^2^ = 0.1979, *P* = 0.0030, Fig. 3e). Antibiotic treatment increased the relative abundance of the family *Lachnospiraceae* (*P* = 0.0001), order *Lachnospirales* (*P* = 0.0003), and family *Butyricicoccaceae* (*P* = 0.0235), and significantly decreased the relative abundance of the orders *Desulfovibrionales* (*P* = 0.0065) and *Lactobacillales* (*P* = 0.0373) and the genera *Desulfovibrio* (*P* = 0.0616) and *Lactobacillus* (*P* = 0.0296; Fig. 3f and Supplementary Fig. 3b). In addition, jejunum villus length was significantly improved in the ANTI group, but intestinal TJP1 was decreased (Fig. 3g and Supplementary Fig. 3c, d).

Afterward, we performed an untargeted metabolomic analysis of serum. OPLS-DA revealed a difference in the metabolite profiles between the HUA and ANTI groups (Fig. 3h). Furthermore, antibiotic treatment significantly decreased UA and metabolites of its synthetic precursors (e.g., xanthosine and inosine), and reversed amino acid depletion (e.g., proline and tryptophan) in HUA (Fig. 3i and Supplementary Fig. 3e). Additionally, a Pearson correlation analysis indicated that the genus *Lactobacillus* had a substantially negative association with the serum UA level (*P* = 0.0112) and a significantly positive correlation with serum the proline level (*P* = 0.0021; Fig. 3j). These results were consistent with our HUA findings, demonstrating the crucial role of *Lactobacillus* in UA degradation. Meanwhile, the variation of serum proline in HUA and ANTI groups, as well as its correlation with *Lactobacillus*, suggested that the serum proline level may be closely related to HUA.

To further demonstrate the alteration of UA metabolism after antibiotic treatment, we tested the expression of UA-producing and transporting-related proteins. Simultaneously, antibiotic treatment significantly decreased the protein expression of intestinal UA reabsorption protein GLUT9 and nucleoside transporter CNT2, but decreased the relative protein expression of the UA excretion transporter ABCG2 (Fig. 3g). Likewise, antibiotic treatment suppressed the elevated expression of hepatic UA production-related proteins in HUA (Fig. 3k). Nephritic UA excretion protein was significantly up-regulated (e.g., OAT1, ABCG2), and UA reabsorption protein was significantly down-regulated (e.g., URAT1, GLUT9; Fig. 3l). Moreover, we also observed a decrease in liver inflammation and serum inflammatory cytokines in the ANTI group (Supplementary Fig. 3f and g).

### Transplantation of HCP diet-derived fecal microorganisms induced HUA in recipient goslings

**Fig. 4:**
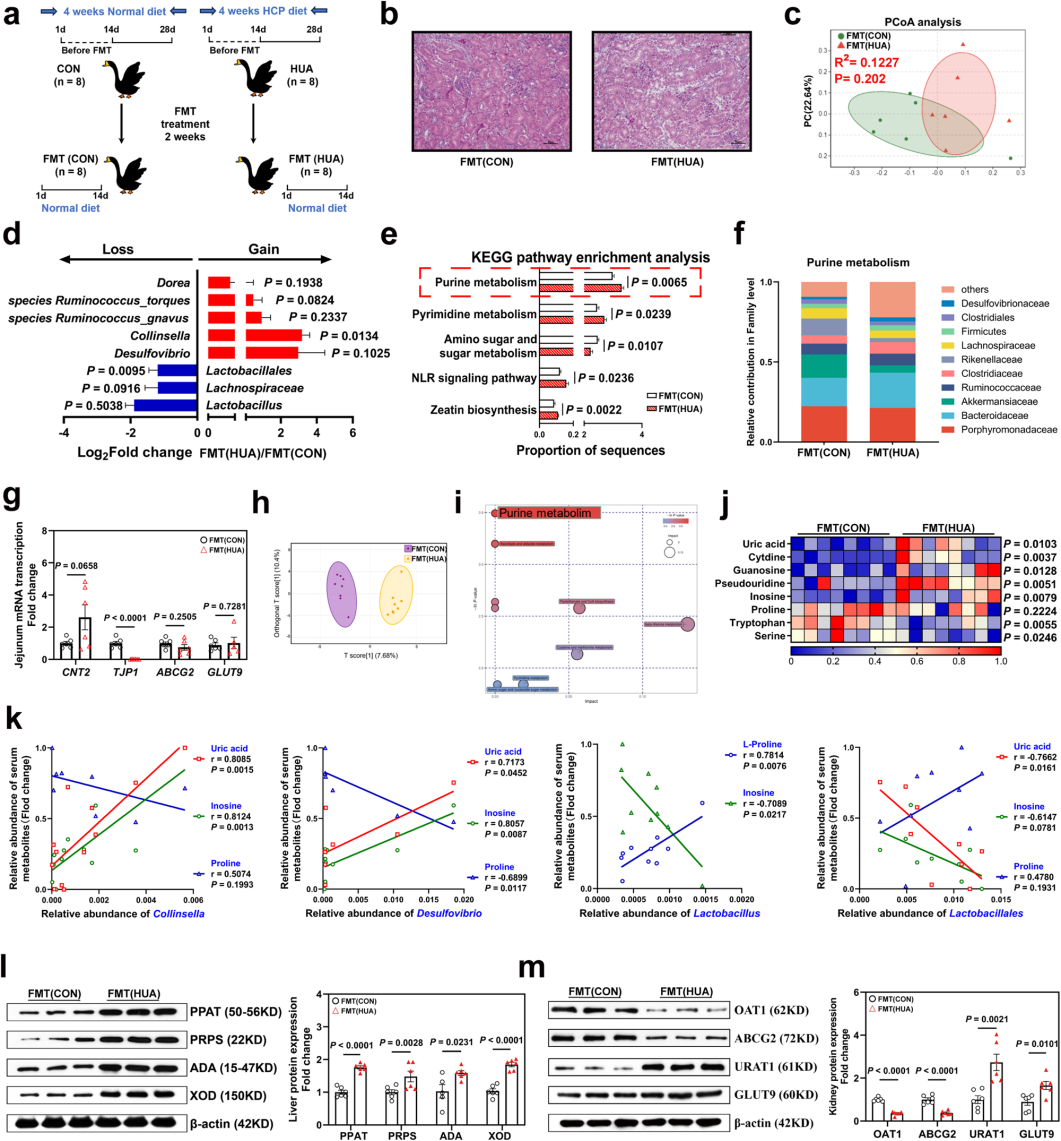
FMT disordered bacteria group and induced the occurrence of HUA through the gut-liver-kidney axis. **a** Fecal microbiota transplantation experimental design. **b** Representative image of H&E staining of kidney sections from FMT(CON) group and FMT(HCP) (×400, *n* = 8). The white part of the peripheral renal tubule in the right figure is the proteinuria protein cast lesions. All scale bars are 50 μm. **c** Principal components analysis of bacteria with 95% confidence regions between the FMT(CON) group (*n* = 6, green) and FMT(HCP) group (*n* = 6, red). **d** The alteration trends of the bacterial relative abundance after HCP diet treatment-derived microbiota treatment (*n* = 6). **e** The abundance of microbial function genes and gene families in the FMT(CON) group (white) and FMT(HCP) group (red), *n* = 6, mean with SEM. **f** Changes in the functional contribution of purine metabolism (top ten bacterial in terms of abundance, Family level). **g** Effect of HCP diet treatment-derived microbiota on the relative expression of nucleoside transport gene (CNT2), gut barrier gene (TJP1), UA excretion genes (ABCG2), and reabsorption genes (GLUT9) in jejunum tissue by RT-PCR analysis (*n* = 6). **h** OPLS-DA of the serum samples (*n* = 8). The purple color represents the FMT(CON) group, while the yellow color FMT(HCP) group. Compounds that were selected through RP and HILIC were analyzed separately. **i** KEGG pathway enrichment differential metabolites between FMT(CON) group and FMT(HCP) group (*n* = 8). The y-axis shows the Ln *P*-value, and the significant pathway with *P* < 0.05 was highlighted. **j** Heatmap of LC-MS data showing serum purine and amino acid metabolite changes under HCP diet (*n* = 8). Increases in metabolite levels are shown in red, whereas blue indicates decreased metabolite. **k** Pearson correlation analysis between *Lactobacillus* relative abundance and serum metabolite relative level. **l** Representative western blotting images and quantification of proteins (PPAT, PRPS, ADA, XOD) in the liver tissue between the FMT(CON) group and FMT(HCP) group (*n* = 6). **m** Representative western blotting images and quantification of proteins (OAT1, ABCG2, URAT, GLUT9) in the kidney tissue between the FMT(CON) group and FMT(HCP) group (*n* = 6). Data with error bars represent mean ± s.e.m. For (**d**, **g**, **j**, **l**, and **m**), data were analyzed by two-tailed unpaired Student’s *t*-test. For e, data were employed for the Wilcoxon rank-sum test. For (**k**), data were employed for computer non-parametric Pearson correlation. FMT (CON): geese received fecal microbiota from CON diet-treated donor geese. FMT (HCP): geese received the fecal microbiota from HCP diet-treated donor geese.

To further investigate the role of the gut microbiota in HUA, FMT was performed on HUA geese (Fig. 4a). Protein, Ca, and P levels in the feces of CON and HUA donor geese were tested to rule out the effects of these components on the alteration of the microbiota in recipient geese (Supplementary Fig. 4a). In general, fecal microbiota transplantation raised the serum UA level and resulted in kidney damage (Supplementary Fig. 4a and Fig. 4b).

A metagenome analysis revealed that the HUA microbiota significantly changed the composition of the intestinal microbiota in the FMT(HUA) group (*R*^2^ = 0.1227, *P* = 0.2020; Fig. 4c). Changes in microbial communities and function genes in the intestine had trends similar to those in the HUA group. Compared to the FMT(CON) group, the abundance of genera *Collinsella* (*P* = 0.0134), *Ruminococcus torques* (*P* = 0.0824), *Ruminococcus gnavus* (*P* = 0.2337), *Dorea* (*P* = 0.1938), and *Desulfovibrio* (*P* = 0.1025) in the FMT (HUA) group were increased, but the relative abundance of the family *Lachnospiraceae* (*P* = 0.0916), order *Lactobacillales* (*P* = 0.0095) and genus *Lactobacillus* (*P* = 0.5038) were reduced (Fig. 4d). This trend was opposite the changes seen in the genus *Lactobacillus* and order *Lactobacillales* abundance in the HUA group induced by the HCP diet (Figs. 4d and 2e). These contradictory findings further suggest that the *Lactobacillus* may act as an antagonistic microbe against HUA in the intestine, thereby leading to its increased abundance in the HUA group but not in the FMT (HUA) group (Supplementary Fig. 4c and Fig. 4d). To survey purine metabolism in the intestine and serum, we performed a KEGG pathway analysis of the intestinal microbiota. KEGG results showed that FMT significantly increased the purine metabolism and the NLR signaling pathway of the intestinal microbiota in recipient geese (Fig. 4e and Supplementary Fig. 4d). A functional contribution analysis revealed that, compared to the FMT(CON) group, the families *Bacteroidaceae*, *Clostridiaceae*, and *Desulfovibrionaceae* showed a greater contribution to purine metabolism in the FMT (HUA) group (Fig. 4f). To further demonstrate the changes in purine metabolism systematically, we performed a serum metabonomic analysis. OPLS-DA revealed distinct serum metabolite profiles in the two groups (Fig. 4h), and the changes in purine metabolism were the most significant (Fig. 4i). The HUA-derived microbiota significantly increased the metabolite level of UA (*P* = 0.0103) and its synthetic precursors, while leading to the depletion of amino acids, such as proline (*P* = 0.2224) and tryptophan (*P* = 0.0055). These results are mainly consistent with the changes in the HUA group (Fig. 2n, Fig. 4j, and Supplementary Fig. 4d). Furthermore, a Pearson correlation analysis indicated that the genus *Lactobacillus* had a negative association with the serum inosine level (*P* = 0.0217) and a significantly positive correlation with the serum proline level (*P* = 0.0076). Meanwhile, the order *Lactobacillales* had a significant negative association with the serum UA level (*P* = 0.0161), the genera *Collinsella* and *Desulfovibrio* had significantly positive correlations with serum UA and inosine levels (*P* < 0.05), and *Desulfovibrio* had a substantial negative association with the serum proline level (*P* = 0.0117; Fig. 4k). These data were consistent with the changes in the microbiota and metabolites in our HUA and ANTI groups, further explaining the probable alleviating function of *Lactobacillus* in HUA and the relevance of *Desulfovibrio* with HUA.

Next, we investigated the UA transportation-related genes in the gut-liver-kidney axis. The mRNA expression of jejunum CNT2 was increased, and those of ABCG2 and TJP1 were decreased in the FMT(HUA) group (Fig. 4g). Consistently, FMT(HUA) treatment significantly increased the expression of UA synthesis-related enzymes PRPS, PPAT, ADA, and XOD in the liver (Fig. 4l), and caused cellular inflammatory infiltration, as well as increased serum IL-1β and TNF-α levels (Supplementary Fig. 4e and f). In the kidney, expression levels of UA excretion transporters OAT1 (*P* < 0.0001) and ABCG2 (*P* < 0.0001) were significantly decreased in the FMT(HUA) group, while those of UA resorption proteins URAT1 (*P* = 0.0021) and GLUT9 (*P* = 0.0101; Fig. 4m) were increased.

Taken together, these findings show that FMT in HUA geese profoundly altered the gut microbial diversity and function in recipient geese. Similar to the results in the HUA group, the HUA-derived microbiota showed a severe disruption of the purine metabolism by the gut microbiota, resulting in an increased load of precursor metabolites of UA generation, meanwhile enhanced liver UA production, and reduced renal UA excretion, finally promoting the development of HUA. Our data demonstrated that *Lactobacillales* and *Lactobacillus* have an antagonistic effect on HUA progression, and serum proline deficiency is involved in the occurrence of HUA.

### LGG and LGG with its metabolites regulated the gut microbiota and alleviated HCP diet-induced HUA through the gut-liver-kidney axis

**Fig. 5:**
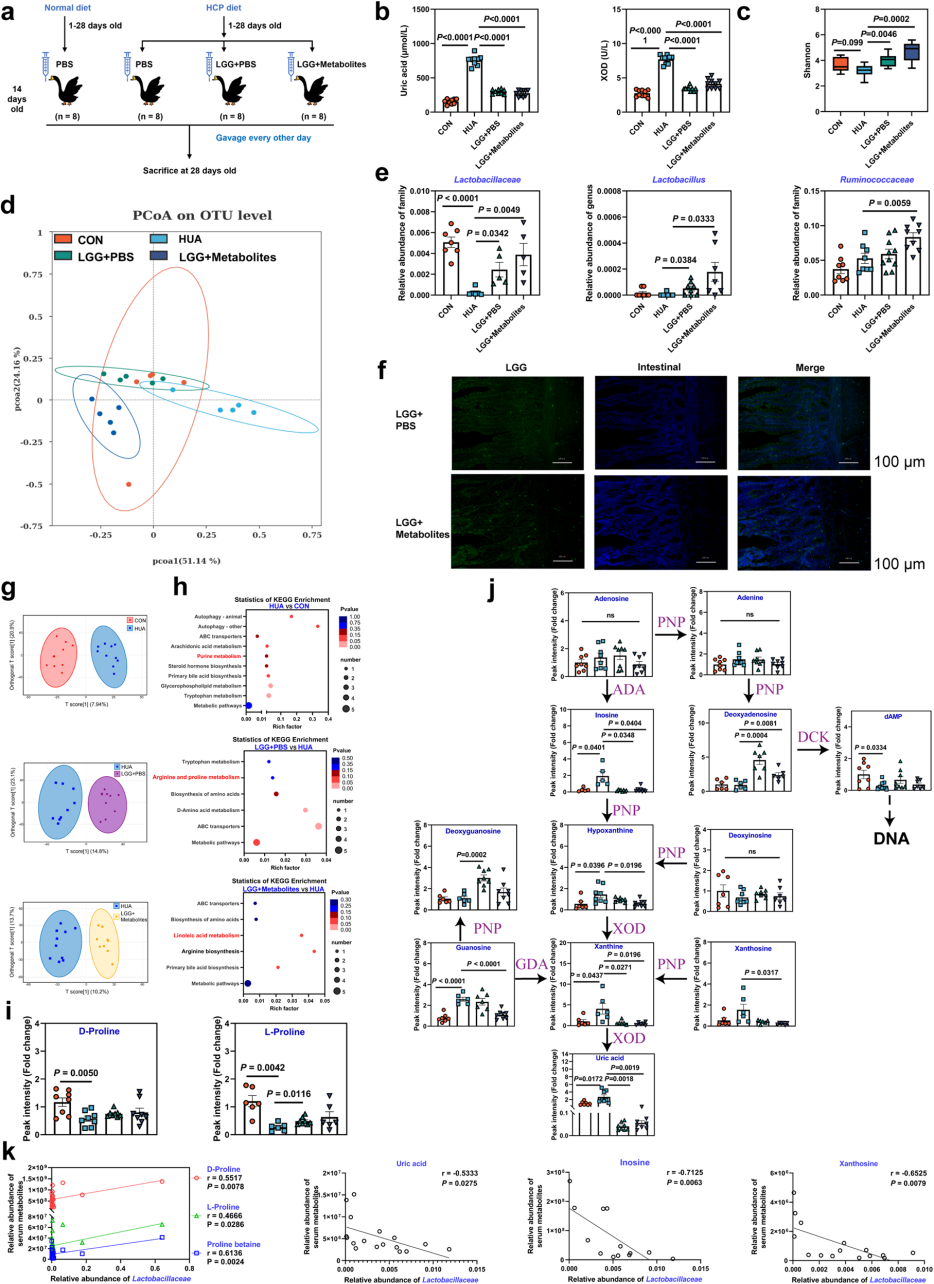
LGG and LGG metabolites alleviate HCP diet-induced HUA through the gut-liver-kidney axis. **a** LGG and LGG metabolites treatment experimental design. LGG + PBS: LGG cells resuspended in PBS, LGG + Metabolites: LGG cells and their metabolites. **b** Effect of LGG and LGG metabolites on the serum UA, and XOD levels in HCP diet-treated geese (*n* = 10). **c** Shannon index of indicated groups based on alpha diversity analysis (*n* = 8). **d** Principal components analysis of bacteria with 95% confidence regions of indicated groups (*n* = 5). **e** The alteration trends of the bacterial relative abundance (*n* = 8). **f** FISH assay was applied to explore the location of LGG in the intestinal (original magnification, ×200, scale bar:100 μm). Green represents LGG (LGG probe, FAM-labeled) and blue represents intestinal cell nucleus (DAPI). **g** Metabolic profiles of serum between two groups are clustered according to OPLS-DA (*n* = 8). The red color represents the CON group, while the blue color represents the HUA group, purple represents the LGG + PBS group, and yellow represents the LGG + Metabolites group. Compounds that were selected through RP and HILIC were analyzed separately. **h** KEGG pathway enrichment differential metabolites between HUA group and CON, LGG + PBS, LGG + Metabolites group (*n* = 8). The x-axis shows the rich factor. **i** The alteration trends of proline relative content between the HUA group and CON, LGG + PBS, LGG + Metabolites group (*n* = 8). **j** Changes in purine pathway metabolites level in LGG and LGG metabolites treatment (*n* = 8). **k** Pearson correlation analysis between gut microbiome relative abundance and serum metabolite relative level. Data with error bars represent mean ± s.e.m. For (**b**, **c**, **e**, **h**, and **i**), data was analyzed by two-tailed unpaired Student’s *t*-test. For (**j**), data were employed for computer non-parametric Spearman correlation.

To further verify the functional role of LGG alone or LGG with its metabolites in HUA, we treated HUA goslings with LGG alone or LGG with its metabolites via oral gavage (Fig. 5a). Both LGG and LGG with its metabolites significantly reduced serum BUN, Cr, XOD, and UA accumulation, and decreased serum IL-1β, IFN-γ, and TNF-α levels (Fig. 5b and Supplementary Fig. 5a). Fluorescence in situ hybridization (FISH) showed that LGG colonized in the jejunum of geese (Fig. 5f). Sequencing analysis of cecal chyme found that both LGG and LGG with its metabolites significantly increased the microbiota richness and uniformity compared with the HUA group (Fig. 5c and Supplementary Fig. 5b). A PCoA analysis showed differences in gut microbiota between the LGG group, the LGG + metabolites group, and the HUA group (*R*^2^ = 0.4403, *P* = 0.0001; Fig. 5d). Sequencing results showed that both LGG and LGG with its metabolites increased the relative abundance of the genus *Lactobacillus*, family *Lactobacillaceae*, and family *Butyricicoccaceae* (Fig. 5e). Meanwhile, the relative abundance of the genus *Butyricicoccus* and family *Ruminococcaceae* also increased (Supplementary Fig. 5c and d). The LEfSe analysis revealed that the family *Lactobacillaceae* in the CON group, genus *Butyricicoccus* in the LGG + PBS group, and both *Lactobacillaceae* and *Butyricicoccus* in the LGG + metabolites group made significant contributions compared to the HUA group (Supplementary Fig. 5e–g). These data demonstrated that gavage with both LGG and LGG with its metabolites alleviated the dysbiosis of intestinal microbes induced by HUA. Next, we performed a metabonomic analysis of serum to investigate purine metabolism in geese. OPLS-DA showed different serum metabolite profiles among groups (CON & HUA groups, HUA & LGG + PBS groups, HUA & LGG + metabolites groups; Fig. 5g). A KEGG enrichment analysis showed that the metabolites in purine metabolism differed between the CON and HUA groups, and metabolites were enriched in arginine and proline metabolism, as well as linolenic acid metabolism after LGG gavage (Fig. 5h). HUA significantly depleted L-proline in serum, and LGG reversed this depletion (Fig. 5i). Additionally, UA and its precursor substances in purine metabolism, such as inosine, HX, and xanthine, were significantly reduced after gavage with LGG alone and LGG with its metabolites, while nucleic acid synthesis pathway-related substances, such as deoxyinosine, deoxyguanosine, and deoxyadenosine, were significantly increased (Fig. 5j). Consistent results were observed with a significant negative correlation between the family *Lactobacillaceae* and serum UA, inosine, and xanthosine while a significant positive correlation was seen with the serum proline level (Fig. 5k). Together, both LGG alone and LGG with its metabolites balanced the dysbiosis of the gut microbiota induced by HUA, significantly increased the abundance of family *Lactobacillaceae*, genus *Lactobacillus*, genus *Butyricicoccus*, and family *Ruminococcaceae*, decreased serum UA and its synthetic precursors, reversed serum proline depletion, and ultimately alleviated HUA in geese.

### LGG alleviated HUA by regulating nucleoside and proline metabolism

**Fig. 6:**
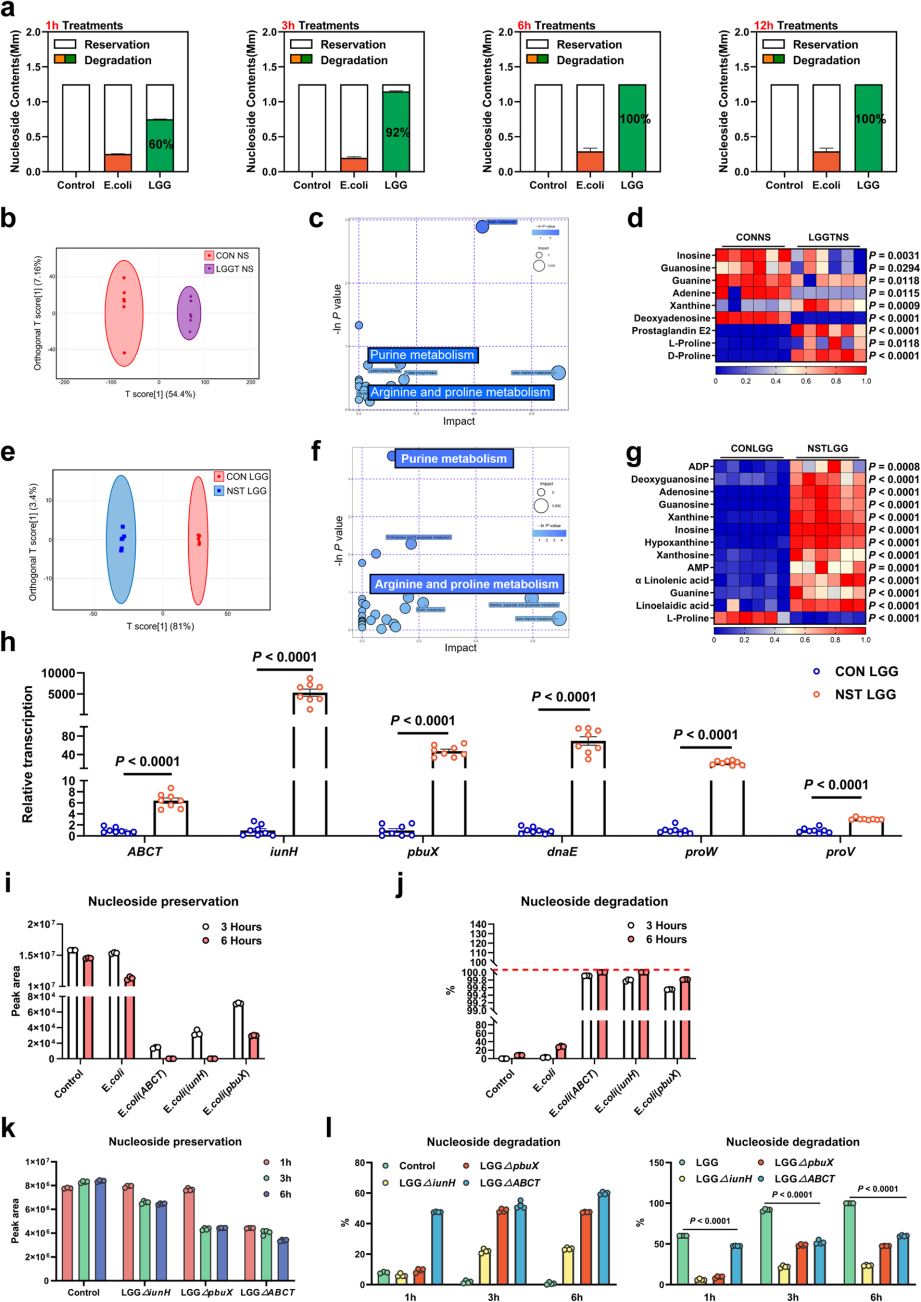
LGG absorbs and degrades nucleosides through nucleoside permease and nucleoside hydrolase, and generates proline, which has the potential to alleviate HUA. **a** Degradation effect of LGG on nucleoside solutions (*n* = 6). The contents of nucleosides (inosine, guanosine) after co-incubation of LGG with nucleoside solution were determined by high-performance liquid chromatography (HPLC). **b** OPLS-DA of the extracellular samples (*n* = 6). The red color represents the CONNS group, while the purple color LGGTNS group. Compounds that were selected through RP and HILIC were analyzed separately. **c** KEGG pathway enrichment differential metabolites between CONNS group and LGGTNS group (*n* = 6). **d** Heatmap of extracellular LC-MS data showing marker metabolite changes between CONNS group and LGGTNS group (*n* = 6). Increases in metabolite levels are shown in red, whereas blue indicates decreased metabolite. **e** OPLS-DA of the intracellular samples (*n* = 6). The red color represents the CONLGG group, while the blue color NSTLGG group. Compounds that were selected through RP and HILIC were analyzed separately. **f** KEGG pathway enrichment differential metabolites between CONLGG group and NTLGG group (*n* = 6). The y-axis shows the Ln *P*-value, and the significant pathway was highlighted. **g** Heatmap of intracellular LC-MS data showing marker metabolite changes between CONLGG group and NSTLGG group. Increases in metabolite levels are shown in red, whereas blue indicates decreased metabolite. **h** Real-time PCR analysis for *ABCT*, *iunH*, *pbuX*, *dnaE*, *proW*, and *proV* in LGG cells under different treatments (*n* = 8). **i,** **j** Effect of heterologous expression of *ABCT*, *iunH*, *pbuX* genes in *E. coli* on the degradation of nucleoside solutions (*n* = 4). Nucleoside (inosine, guanosine) preservation was determined by HPLC. The nucleoside degradation rates were obtained by conversion from the results of the standards. **k,** **l** Degradation effects of *iunH*, *pbuX*, and *ABCT* knockouts in LGG on nucleoside solutions (*n* = 4), control: inosine, guanosine. The nucleoside degradation rates were obtained by conversion from the results of the standards. Data with error bars represent mean ± s.e.m. For (**a**, **d**, **g**, and **h**), data were analyzed by two-tailed unpaired Student’s *t*-test. CONNS: control nucleoside solution, *LGGTNS* LGG-treated nucleoside solution, *CONLGG* control LGG; *NSTLGG* nucleoside solution-treated LGG, yxjA purine nucleoside transport protein, *ABCT* ABC-type multidrug transport system, ATPase and permease component; *iunH* Inosine-uridine nucleoside N-ribohydrolase, *pbuX* xanthine permease, *dnaE* DNA polymerase III subunit alpha, *proW* proline transport system permease protein, *proV*: proline transport system ATP-binding protein.

To further elucidate the function of LGG, we performed a whole-genome sequencing analysis. Genes involved in nucleoside transport and degradation, such as *ABCT*, *iunH*, purine-nucleoside phosphorylase *deoD*, and *pbuX* were identified (Supplementary Fig. 6d and e). The proline synthesis gene (*proC*) and transport-related genes (*proV*, *proW*, *proX*) in the LGG genome also demonstrated the function of LGG in improving proline metabolism in HUA (Supplementary Fig. 6e). To further illustrate the potential function of LGG in UA degradation, we used *E. coli* as a comparison for incubation with inosine, which is the intermediate metabolite of UA in the intestine. HPLC results showed that the nucleoside degradation rate in the LGG group reached up to 90% after incubation for 3 h, which was significantly higher than those in the *E. coli* and control groups (Fig. 6a).

To comprehensively investigate the metabolic changes of LGG in a high nucleoside condition, we tested the metabolic changes in nucleoside solutions (inosine and guanosine) with and without LGG treatment (CONNS and LGGTNS groups). OPLS-DA and KEGG analysis showed that the metabolite profiles in extracellular supernatant of the two groups were different, and metabolites were enriched in the purine metabolic pathway (Fig. 6b and c). Heatmaps of differential metabolites showed that LGG significantly reduced UA synthesis precursors, such as inosine, guanosine, guanine, and xanthine, while increasing proline content in the supernatant (Fig. 6d). These findings, along with the changes in serum proline in our animal results, suggested that LGG could generate proline both in animal intestines and in vitro. Next, we further investigated the intracellular metabolic changes in LGG with and without nucleoside incubation (inosine and guanosine; CONLGG and NSTLGG groups). Consistently, OPLS-DA analysis of intracellular metabolite profiles in two groups showed that intracellular UA precursor substances, such as inosine, guanosine, guanine, xanthine, and HX, were significantly increased, and proline was significantly reduced. These findings, along with the result of whole-genome analysis, suggested that LGG may generate proline via the *proC* (pyrroline-5-carboxylate reductase) gene, and transport it from an intracellular space to an extracellular space via *proV* and *proW* genes (Fig. 6e–g).

In summary, we conclude that LGG could positively absorb and degrade nucleosides while producing and excreting proline in the presence of nucleoside. We hypothesized that high-nucleoside conditions could promote the expression of nucleoside transport genes, thereby activating the purine metabolism pathway, as well as the arginine and proline metabolism pathways in LGG, according to the OPLS-DA and KEGG results. To test this hypothesis, the relative transcription expression of LGG under nucleoside conditions was measured. Our results showed that the LGG genes *ABCT*, *iunH*, and *pbuX* were significantly upregulated, and *proW* and *proV* were also significantly upregulated in nucleoside-solution-treated LGG (Fig. 6h). The significant up-regulation of DNA synthase (*dnaE*) and adverse changes in intra- and extracellular deoxyadenosine of LGG demonstrated that LGG may synthesize its DNA by converting nucleosides to deoxynucleosides and deoxynucleotides (Fig. 6d, g and h). To further demonstrate the function of these genes in LGG, we examined heterologous expression in *E. coli* and determined the nucleoside degradation rates in *E. coli* by HPLC. Heterologous expression of *ABCT*, *iunH*, and *pbuX* in E. *coli* showed that *ABCT*, *iunH*, and *pbuX* genes could degrade nucleosides, and more than 90% of nucleosides were degraded in 3 h. Among them, heterologous expression of the *ABCT* gene showed slightly more rapid degradation than that of other genes (Fig. 6i and j). Next, we performed gene knockout of the *ABCT, iunH*, and *pbuX* genes, respectively in LGG, and incubated with nucleoside solution to demonstrate the functions of these three genes in purine metabolism. HPLC results showed that the nucleoside degradation rates of LGG_∆_*ABCT*, LGG_Δ_*pbuX*, and LGG_∆_*iunH* were significantly lower than that of LGG (*P* < 0.0001, Fig. 6k and l). Notably, LGG_Δ_*iunH* has the most significant suppression of degradation function, compared with the other two deletion strains. Collectively, these data suggest that LGG alleviates HUA through absorption and degradation of nucleosides by the *ABCT*, *iunH*, and *pbuX* genes, while, generating and excreting proline. However, additional studies will be needed to elucidate whether LGG metabolites, such as proline, and other substances could alleviate HUA.

### LGG metabolites and proline alleviated the dysfunction of the intestine, liver, and kidney in HUA

**Fig. 7:**
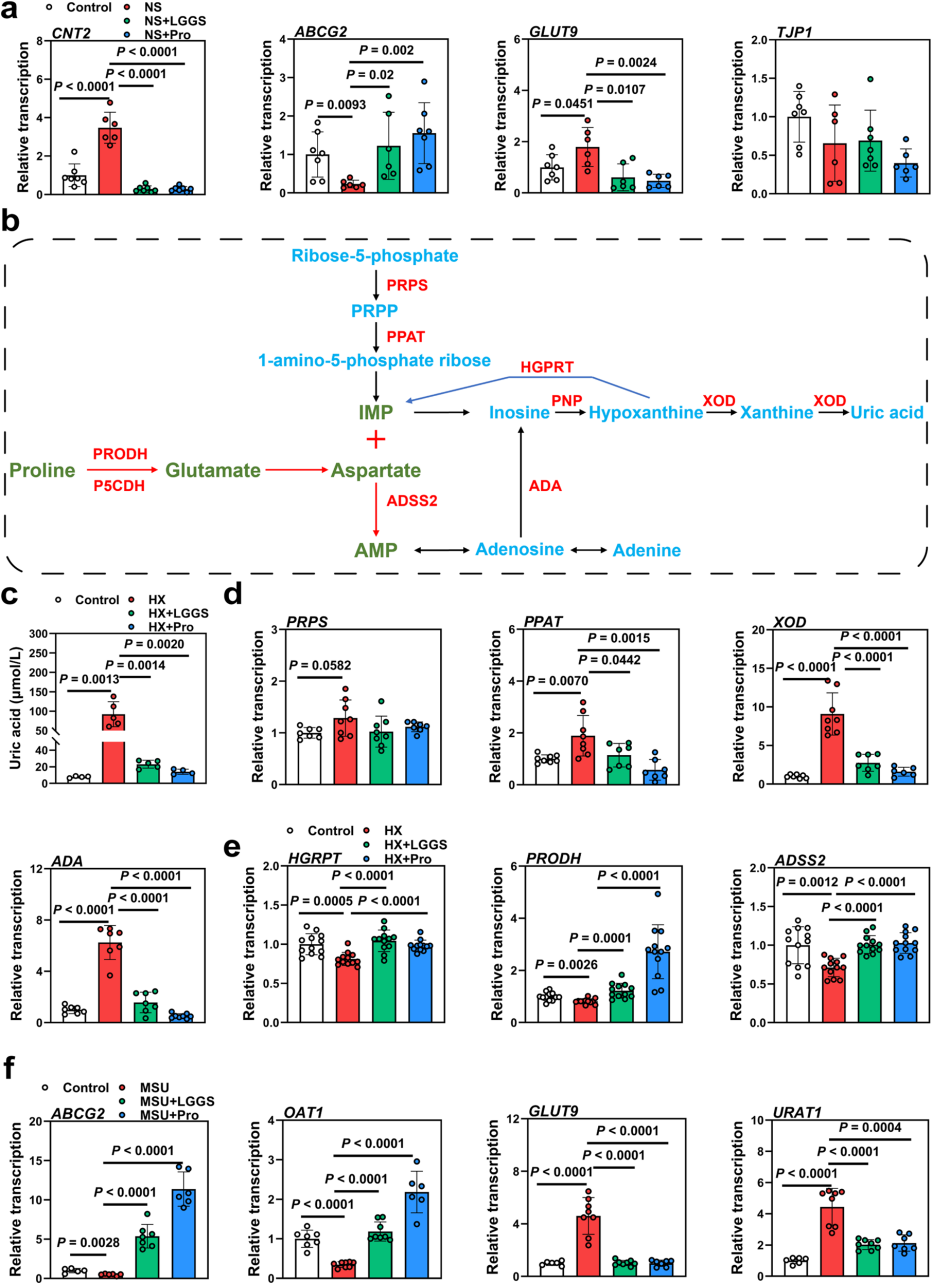
LGG metabolites or proline can alleviate the dysfunction of the intestine, liver, and kidney. **a** Effect of LGG metabolites or proline on the relative expression of nucleoside transport gene (*CNT2*), gut barrier gene (*TJP1*), UA excretion genes (*ABCG2*), and reabsorption genes (*GLUT9*) in IPEC-J2 cell by RT-PCR analysis (*n* = 8). **b** proline metabolism and purine metabolism pathways. **c** Effect of LGG metabolites or proline on the UA levels in HX treated Hep-G2 cell (*n* = 5). **d** Effect of LGG metabolites or proline on the relative expression of UA production indicated genes (*PPAT*, *PRPS*, *ADA*, XOD) in Hep-G2 cell by RT-PCR analysis (*n* = 8). **e** Effect of LGG metabolites or proline on the relative expression of UA reduction indicated genes (*HGPRT*, *ADSS2*, *PRODH*) in Hep-G2 cell by RT-PCR analysis (*n* = 12). **f** Effect of LGG metabolites or proline on the relative expression of UA excretion genes (OAT1, ABCG2) and reabsorption genes (*URAT1*, *GLUT9*) in BHK cell by RT-PCR analysis (*n* = 6). Data with error bars represent mean ± s.e.m. For (**a**, **b**, and **c**), data were analyzed by two-tailed unpaired Student’s *t*-test. *NS* nucleoside solution, *LGGS* LGG metabolites solution. *HX* hypoxanthine, *MSU* Monosodium urate, *CNT2* Concentrative nucleoside transporter 2, *TJP1* Tight junction protein 1, *ABCG2* ATP-binding cassette sub-family G member 2, *GLUT9* Glucose transporter 9, *OAT1* Organic anion transporter 1, *URAT1* Urate transporter 1, *PPAT* Phosphoribosyl pyrophosphate amidotransferase, *PRPS* Phosphoribosyl pyrophosphate synthetase, *ADA* Adenosine deaminase, *XOD* Xanthine oxidase dehydrogenase, *HGPRT* Hypoxanthine-Guanine phosphoribosyltransferase, *ADSS2* Adenylosuccinate synthase 2, *PRODH* Proline dehydrogenase.

The studies described above found that serum proline was significantly reduced in HUA geese and significantly increased in the supernatant of nucleoside-treated LGG, but further investigation will be needed to determine whether LGG metabolites and proline play a role in UA degradation (Fig. 2n and 6d). Therefore, we designed in vitro experiments using mammal intestinal (IPEC-J2), liver (Hep-G2), and kidney (BHK) cell lines to demonstrate whether LGG metabolites or proline could play roles in nucleoside degradation. A gradient experiment was performed to determine the concentration and treatment time of nucleoside, proline, and LGG supernatant (Supplementary Fig. 7a, b). In intestine cell lines, both LGG metabolites and proline down-regulated intestinal UA reabsorption protein GLUT9 and nucleoside transporter CNT2 mRNA expression levels, and upregulated UA excretion transporter ABCG2 and TJP1 mRNA expression, suggesting that both LGG metabolites and proline increased UA excretion to the lumen and decreased nucleoside transport to serum (Fig. 7a). To illustrate the role of LGG metabolites and proline in UA synthesis in the liver, HX, the precursor of UA, was used in the hepatic HUA experiment (Fig. 7b). Concentrations and treatment times of HX, LGG metabolites, and proline in Hep-G2 cells were determined by gradient assays (Supplementary Fig. 7c). HX treatment significantly increased UA levels in HX-treated Hep-G2 cells. HX also upregulated the mRNA expression of UA synthesis-related enzymes PRPS, PPAT, ADA, and XOD (Fig. 7c, d), and significantly down-regulated mRNA expression of UA synthesis-reversal enzymes hypoxanthine-guanine phosphoribosyltransferase (HGPRT) and adenylosuccinate synthase 2 (ADSS2) (Fig. 7e), while LGG metabolites and proline significantly reversed these changes. We also observed up-regulation of the proline dehydrogenase (PRODH) gene in LGG metabolites and proline treatment groups, which demonstrated that proline is critically involved in the alleviation of HUA (Fig. 7e). We next tested the effects of LGG metabolites and proline in BHK cells by treatment with monosodium urate (MSU), which is the main form of UA in the kidney (Supplementary Fig. 7d). As a result, both LGG metabolites and proline significantly upregulated the mRNA expression of UA excretion transporter OAT1 and ABCG2, and down-regulated the mRNA expression of UA resorption proteins URAT1 and GLUT9 (Fig. 7f). These results suggested that LGG metabolites could alleviate the dysfunction of the intestine, liver, and kidney in HUA, and proline is probably the main metabolite responsible for this function. These results suggested that LGG metabolites could alleviate the dysfunction of the intestine, liver, and kidney in vitro induced by HUA, and proline plays a pivotal role in LGG metabolites.

### LGG in the diet alleviated HCP diet-induced HUA through the gut-liver-kidney axis

**Fig. 8:**
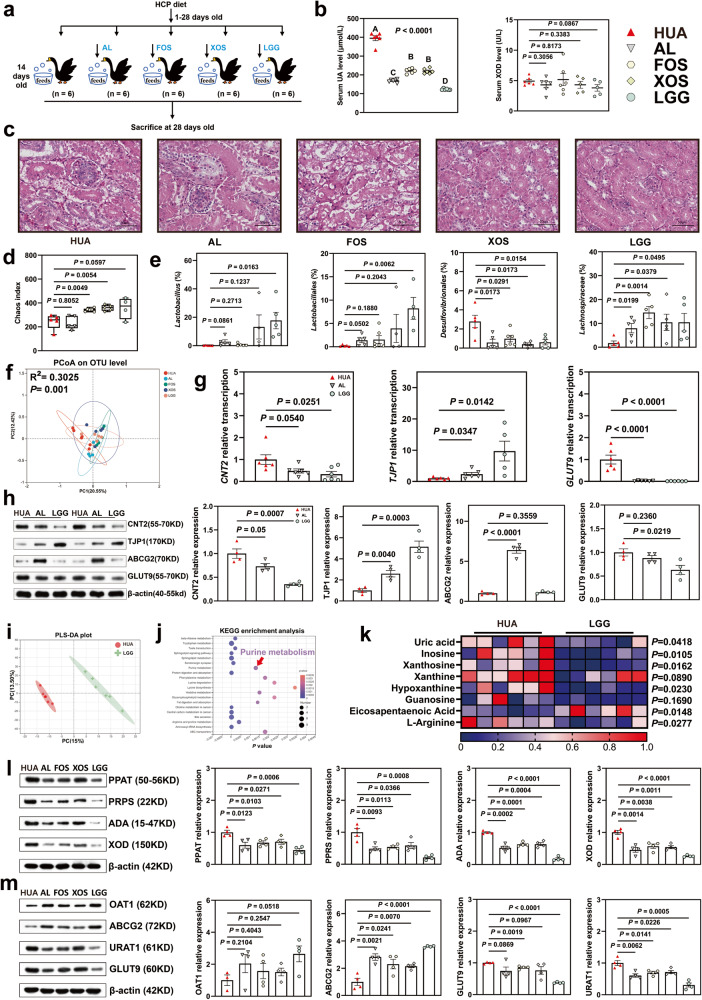
LGG treatment adjusts gut flora and alleviates HCP diet-induced HUA through the gut-liver-kidney axis. **a** LGG and prebiotics treatment experimental design. *AL* Allopurinol, *FOS* fructo-oligosaccharide, *XOS* xylo-oligosaccharide. **b** Effect of LGG, AL, and prebiotics on the serum UA, and XOD levels in HCP diet-treated geese (*n* = 6). **c** Representative image of H&E staining of kidney sections from indicated groups (×400, scale bar: 50 μm, *n* = 6). The white part of the peripheral renal tubule in the right figure is the proteinuria protein cast lesions. All scale bars are 50 μm. **d** Chao index of indicated groups based on alpha diversity analysis (*n* = 6). **e** The alteration trends of the bacterial relative abundance (*n* = 6). **f** Principal components analysis of bacteria with 95% confidence regions of indicated groups (*n* = 6). **g** Effect of LGG, AL treatment on the relative expression of nucleoside transport gene (CNT2), gut barrier gene (TJP1), and UA reabsorption indicated genes (GLUT9) in jejunum tissue by RT-PCR analysis (*n* = 6). **h** Representative western blotting images and quantification of proteins (CNT2, TJP1, ABCG2, GLUT9) in the jejunum tissue between the HUA group, AL group, and LGG group (*n* = 4). **i** PLS-DA of the serum samples (*n* = 6). The red color represents the HUA group, while the green color LGG group. Compounds that were selected through RP and HILIC were analyzed separately. **j** KEGG pathway enrichment differential metabolites between HUA group and LGG group (*n* = 6). The x-axis shows the Ln *P*-value. **k** Heatmap of LC-MS data showing serum purine, lipid acid, and amino acid metabolite changes under HCP diet (*n* = 6). Increases in metabolite levels are shown in red, whereas blue indicates decreased metabolite. **l** Representative western blotting images and quantification of proteins (PPAT, PRPS, ADA, XOD) in the liver tissue of indicated groups (*n* = 4). **m** Representative western blotting images and quantification of proteins (OAT1, ABCG2, URAT, GLUT9) in the kidney tissue of indicated groups (*n* = 4). Data with error bars represent mean ± s.e.m. For (**b**, **d**, **e**, **g**, **h**, **k**, **l**, and **m**), data were analyzed by two-tailed unpaired Student’s *t*-test.

To further validate the antagonistic effect of LGG on HUA, we added LGG freeze-dried powder to the diet of a HUA goose model, and used allopurinol (AL), FOS, and XOS as positive controls (Fig. 8a).

Our results showed that AL, FOS, XOS, and LGG greatly reduced serum UA and BUN contents in geese, while alleviating renal injury. Particularly, LGG decreased the activity of serum XOD (Fig. 8b, c, and Supplementary Fig. 8a).

Compared with the HUA group, LGG treatment significantly balanced the dysbiosis of the gut microbiota, and a PCoA analysis indicated that LGG significantly changed the intestinal flora composition (*R*^2^ = 0.3025, *P* = 0.0010; Fig. 8d and f). Moreover, LGG significantly increased the relative abundance of the genus *Lactobacillus* (*P* = 0.0163), order *Lactobacillales* (*P* = 0.0062), and family *Lachnospiraceae* (*P* = 0.0495), while decreasing the relative abundance of the order *Desulfovibrionales* (*P* = 0.0154, Fig. 8e). These results echo our previous finding that *Desulfovibrionales* is involved in the development of HUA and *Lactobacillus* may have an antagonistic effect on HUA.

To further determine whether LGG in the diet altered UA metabolism in the host, we analyzed serum metabolites in the HUA and LGG groups. PLS-DA analysis showed that the HUA and LGG groups had different metabolite profiles (Fig. 8i). KEGG enrichment analysis showed that LGG significantly altered the purine metabolism pathway (Fig. 8j). Levels of UA and UA precursor metabolites were significantly decreased in the LGG group, and the level of eicosapentaenoic acid was up-regulated (Fig. 8k).

The jejunum morphology and western blotting (WB) results showed that LGG significantly improved the intestinal epithelial mucosal integrity, and both the villus height of the intestine and TJP1 protein expression were increased (Fig. 8g, h and Supplementary Fig. 8b, c). HE staining of the liver showed that LGG treatment alleviated inflammation and infiltration (Supplementary Fig. 8d). Meanwhile, levels of IL-1β, IFN-γ, and TNF-α were significantly reduced in the liver of the LGG group (Supplementary Fig. 8e).

Next, we measured the UA metabolic proteins in the gut-liver-kidney axis by RT-PCR and WB. Both AL and LGG treatment significantly down-regulated GLUT9 and CNT2 at both the mRNA and protein levels, and LGG up-regulated TJP protein expression, which means that LGG promotes UA excretion and inhibits its reabsorption in the intestine (Fig. 8g and h). In the liver, UA synthesis-related enzymes PRPS, PPAT, ADA, and XOD were significantly down-regulated at the mRNA and protein levels under treatment with AL, FOS, XOS, and LGG (Fig. 8l). LGG treatment significantly up-regulated the nephritic UA excretion transporter OAT1 and ABCG2, and down-regulated the resorption protein URAT1 and GLUT9 at both the mRNA and protein levels (Fig. 8m). LGG had a stronger alleviation effect on these purine-related genes and protein expressions, compared with AL and prebiotics. Collectively, LGG treatment altered the gut microbiota composition of HUA geese, increased the abundance of the genus *Lactobacillus* and order *Lactobacillales* abundance, and decreased the abundance of the order *Desulfovibrionales* in the intestine. Meanwhile, LGG decreased the precursors of UA in serum, increased UA excretion and inhibited its reabsorption in the gut and kidney, and attenuated UA synthesis in the liver.

### The abundance of intestinal *Lactobacillus* in the HUA population decreased, and the serum UA synthesis precursor increased

**Fig. 9:**
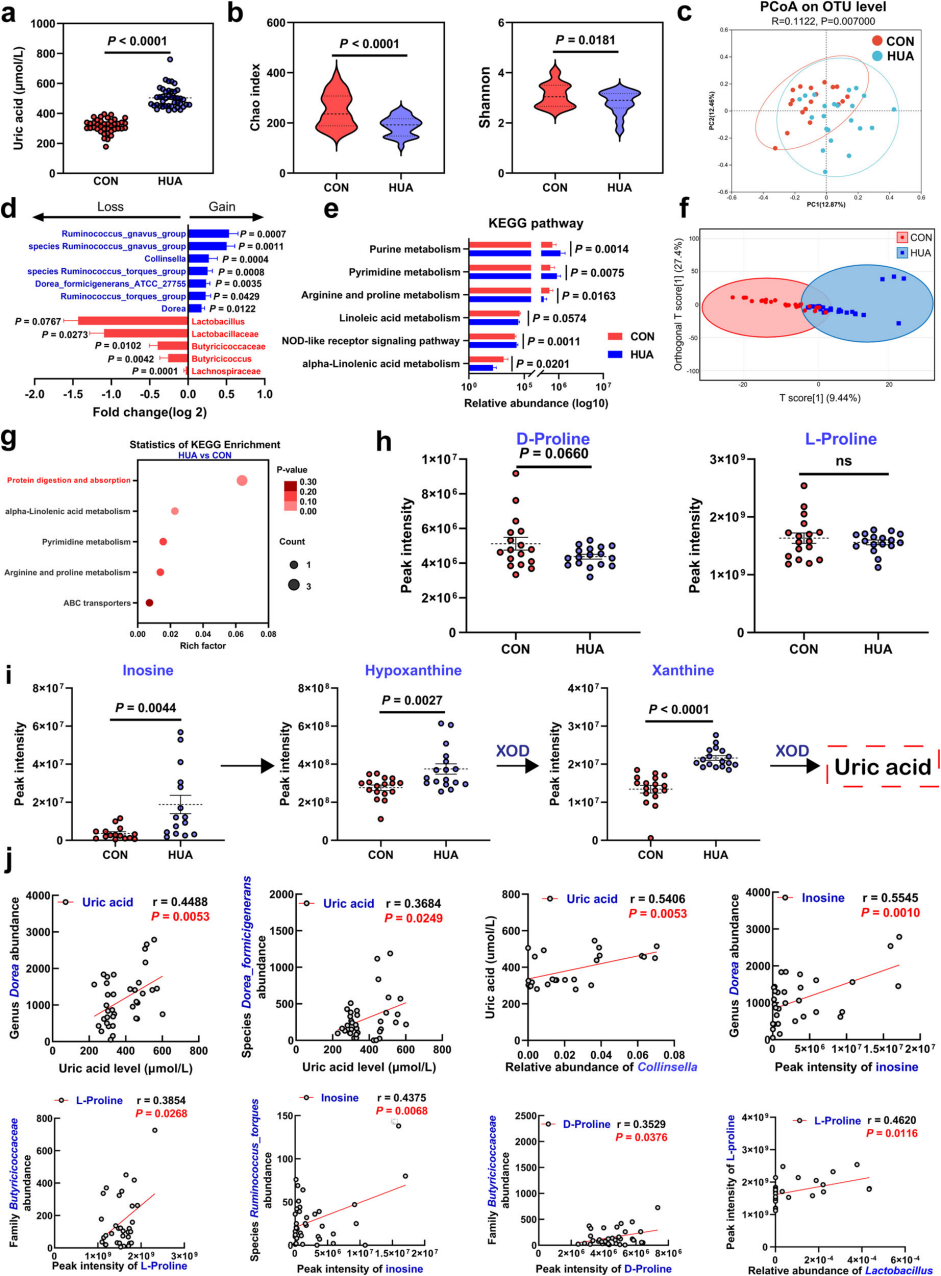
The abundance of intestinal Lactobacillus in the HUA population decreased, and the serum uric acid synthesis precursor increased. **a** Level of serum UA between CON group (*n* = 40) and HUA group (*n* = 43). **b** Chao index of indicated groups based on alpha diversity analysis (CON, *n* = 32; HUA, *n* = 33). **c** Principal components analysis of bacteria with 95% confidence regions of indicated groups. The red color represents the CON group (*n* = 32), while the blue color HUA group (*n* = 33). **d** The alteration trends of the bacterial relative abundance (CON, *n* = 32; HUA, *n* = 33). **e** Abundance profile of the kegg pathway obtained based on PICRUSt prediction analysis. **f** OPLS-DA of the serum samples. The red color represents the CON group (*n* = 25), while the blue color HUA group (*n* = 25). Compounds that were selected through RP and HILIC were analyzed separately. **g** KEGG pathway enrichment differential metabolites between the CON group (*n* = 25) and HUA group (*n* = 25). The x-axis shows the rich factor. **h** The alteration trends of proline relative content between CON (*n* = 17) and HUA group (*n* = 17). **i** Serum purine pathway metabolites levels in CON (*n* = 17) and HUA group (*n* = 17). **j** Pearson correlation analysis between gut microbiome relative abundance and serum uric acid and metabolites relative level. Data with error bars represent mean ± s.e.m. For (**a**, **b**, **e**, **h**, and **i**), data was analyzed by two-tailed unpaired Student’s *t*-test. For (**d**), data were employed for computer non-parametric Spearman correlation.

Next, we randomly collected serum and feces from healthy controls and HUA patients. HUA patients have extremely high serum UA levels (503.4 ± 69.3 μmol/L), compared with those in the healthy population (315.9 ± 46.3 μmol/L) (Fig. 9a). Fecal microbial sequencing analysis revealed that fecal microbial abundance and homogeneity were significantly reduced in HUA patients (Fig. 9b and Supplementary Fig. 9a). PCoA analysis showed a significant difference in fecal microbiota between the HUA and healthy groups (*R*^2^ = 0.0012, *P* = 0.0070; Fig. 9c). Meanwhile, the abundance of the family *Lactobacillaceae* (*P* = 0.0273), genus *Lactobacillus* (*P* = 0.0767), family *Butyricicoccaceae* (*P* = 0.0102), genus *Butyricicoccus* (*P* = 0.0042), and family *Lachnospiraceae* (*P* = 0.0001) were significantly decreased in HUA patients, while the abundance of the genus *Dorea* (*P* = 0.0122), *Ruminococcus_torques_group* (*P* = 0.0429), *Ruminococus_gnavus_group* (*P* = 0.0007)*, Collinsella* (*P* = 0.0004), species *Dorea_formicigenerans_ATCC_27755* (*P* = 0.0035), species *Ruminococcus_torques_group* (*P* = 0.0008), and species *Ruminococus_gnavus_group* (*P* = 0.0008) were significantly increased (Fig. 9d and Supplementary Fig. 9b). LEfSe analysis showed that *Dorea* and *Ruminococcus_torques_group* had the highest contributions in the flora of HUA patients (Supplementary Fig. 9c). Based on the results of a Phylogenetic Investigation of Communities by Reconstruction of Unobserved States prediction analysis, the abundance profile of the KEGG pathway revealed that purine metabolism in intestinal microbes was significantly increased, while arginine and proline metabolism were considerably decreased (Fig. 9e). Subsequently, we performed serum metabolomics analyses for the HUA and healthy groups. OPLS-DA revealed that the serum metabolite profiles in the healthy and HUA groups were different (Fig. 9f). A pathway enrichment analysis demonstrated that these metabolites primarily belonged to 5 pathways: protein digestion and absorption, alpha-linolenic acid metabolism, pyrimidine metabolism, arginine metabolism, and proline metabolism, and ABC transporters, among which protein digestion and absorption appears to be the key node (Fig. 9g). Particularly, serum proline was decreased in the HUA group, which is consistent with our results in geese (Fig. 9h). Additionally, UA synthesis precursors (such as inosine, HX, and xanthine) were significantly increased in the HUA group (Fig. 9i). A Pearson correlation analysis indicated that the genera *Dorea* and *Collinsella*, and species *Dorea_formicigenerans_ATCC_27755* were significantly positively correlated with the serum UA level, the genus *Dorea* and species *Ruminococcus_torques_group* were significantly positively correlated with the serum inosine level, and the genus *Lactobacillus* and family *Butyricicoccaceae* were significantly positively correlated with the serum proline level (Fig. 9j). These data consistently proved our results in geese, demonstrating that *Lactobacillus* plays a pivotal role in HUA and that the serum proline level can serve as a biomarker for HUA occurrence and alleviation.

### While a mouse HUA model did not show elevated serum UA levels, mouse HUA was associated with *Lactobacillus*

To verify the feasibility of using mice in HUA modeling and the effects of *Lactobacillus* in this model, we performed a HUA modeling experiment in mice. The HUA model was constructed by injecting oteracil potassium and HX intraperitoneally in mice (Supplementary Fig. 10a). Although there was a significant decrease in terminal weight and a significant increase in knee thickness in HUA mice, the serum UA level in the HUA group did not exceed the threshold in HUA mice (Supplementary Fig. 10b and c). A pathway analysis and heatmap showed that serum metabolites related to purine and proline metabolism were not significant (Supplementary Fig. 10d, e, and f). Notably, microbial richness and diversity were significantly increased in the HUA group compared to those in the CON group (Supplementary Fig. 10g). A PCoA analysis showed a significant separation of fecal microbiota in the HUA and CON groups (*R*^2^ = 0.0012, *P* = 0.0010; Supplementary Fig. 10h). As in humans and geese (Fig. 2e and Fig. 9d), the abundance of the genus *Lactobacillus* is a significant characteristic in the mouse model, and the family *Lactobacillaceae* and genus *Lactobacillus* were the top contributors in the CON group (Supplementary Fig. 10i and k). However, the abundance of the family *Lactobacillaceae* and genus *Lactobacillus* in the HUA group were significantly decreased compared to those in the CON group (Supplementary Fig. 10j). In summary, although HUA modeling did not result in any changes to the serum UA level in this mouse model, there was a significant reduction in the abundance of the family *Lactobacillaceae* and genus *Lactobacillus* due to HUA. This finding was consistent with our findings in geese and humans.

## Discussion

**Fig. 10:**
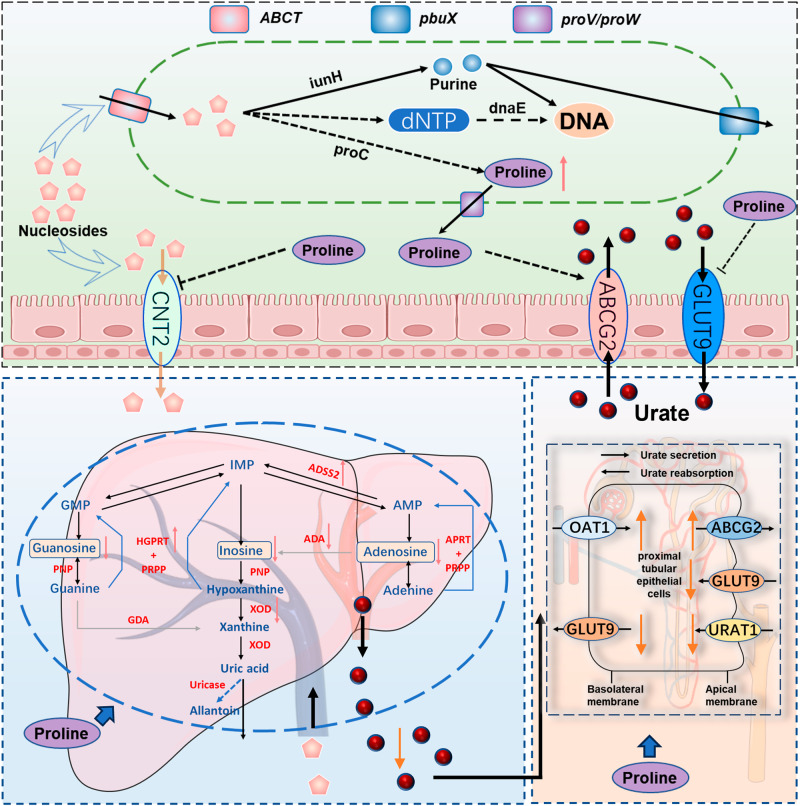
Working hypothesis. The results may indicate that *Lactobacillus rhamnosus* GG degrades nucleosides through absorption, produces metabolites such as proline, increases intestinal uric acid excretion, reduces intestinal nucleoside transport, and reduces liver Uric acid production, restores kidney uric acid excretion, relieves HUA by gut-liver-kidney axis. *ABCT* ABC-type multidrug transport system, ATPase and permease component; *iunH* Inosine-uridine nucleoside N-ribohydrolase, *pbuX* xanthine permease, proW proline transport system permease protein, proV proline transport system ATP-binding protein, *GLUT9* Glucose Transporter 9, UA reabsorption transporter; *CNT2* concentrative nucleoside transporter type, *ABCG2* ATP Binding Cassette Transporter G2, UA excretion transporter, *OAT1* Organic Anion Transporter 1, *XOD* xanthine oxidase.

HUA is increasingly being used as risk factor for gout and other metabolic diseases in different regions of the world. In this study, we established a novel model of HUA in geese, and found a significant correlation between the gut microbiota and HUA. We demonstrated the protective mechanism of LGG in the HUA gosling model. In HUA geese, LGG balanced the microbiota dysbiosis, reduced nucleoside absorption, and increased uric acid excretion through the gut-liver-kidney axis, reducing hepatic and nephric inflammation, and improving serum proline deletion, thereby alleviating HUA (Fig. 10). These findings indicate that goose is a feasible model for studying HUA and LGG is a promising therapeutic dietary supplement for the alleviation of HUA.

In our study, we demonstrated the validity of using goose as a novel model for HUA. The susceptibility to gout in poultry, particularly goslings, renders them highly vulnerable, with a mortality rate of up to 70%^34^. Geese show gout symptoms similar to those in humans, such as effusion and swelling of the joints, as well as urate crystals in particular tissue fluid. Compared with mice, both geese and humans lack uricase in purine metabolism, which is especially important for the formation of HUA^7,8^. In contrast to geese, humans can excrete a portion of the ammonia produced by protein degradation through the intestinal-hepatic urea cycle, therefore reducing the production of UA. Consequently, geese are more susceptible to HUA due to lack of the enzyme arginase, which makes them unable to synthesize urea. In the mouse model, the presence of both uricase and arginase means that the mouse is not susceptible to HUA and it is difficult to induce HUA with diet. However, HUA in goslings can be primarily diet-induced, which is similar to the causes of HUA in humans. However, current mouse models of HUA are generally one of two types: genetically modified models and environmentally induced mouse models that involve oral uricase inhibitors^35,36^. Notably, the UA thresholds for HUA in geese and humans are identical, at around 410–420 μmol/L^37,38^. In mice, the circulating urate concentration is much lower than that in humans, and different serum urate concentrations have been reported in different mouse models^9^. In summary, the goose has gout symptoms similar to those in humans, and it may be an ideal animal model for studying HUA.

Gut microbes have been shown to be associated with host growth, immunity, metabolism, and disease^39,40^. Based on the HUA model in goose, we demonstrated that an HCP diet could cause HUA through the elevation of serum UA, dysbiosis of gut microbes, enhancement of systemic purine metabolism and swelling of the joints. The administration of antibiotic and FMT verified the correlation between gut microbes and HUA. The higher abundance of *Lactobacillus* at the genus level and LGG at the species level in HUA goslings captured our attention. Since previous studies have also observed an increase in the *Lactobacillus* genus in HUA goslings, and considering that the LAB family plays a role in UA degradation, we speculate that LGG may contribute to UA reduction in HUA goose^31,41–43^. In addition, we also found that the abundance of the genera *Desulfovibrio* and *Collinsella* in HUA and FMT goslings were significantly increased and a significant positive correlation was observed between these two genera and UA elevation, demonstrating that the aggravation of HUA may play a role in the higher abundance of the genera *Desulfovibrio* and *Collinsella*. The increase in the genus *Desulfovibrio* is known to be linked to strong co-excluding associations with the occurrence of metabolic syndrome in the host^44,45^. Previous studies suggested that serum UA is determined by the net balance, that is, by purine reabsorption and secretion in the kidney and gut. We found that hepatic purine metabolism also plays an important role^9,46^. In our study, protein expression of urate transporters in the gut and kidney, as well as UA synthesis genes in the liver, demonstrated that UA was systematically accumulated through the gut-liver-kidney axis. These data suggest that gut dysbiosis has a consequential effect on HUA through the gut-liver-kidney axis.

Numerous studies have reported that the family *Lactobacillaceae* could reduce serum UA and modulate the dysbiosis of the gut microbiota in mice^42^. However, a more comprehensive understanding of the bacterial degradation mechanism should be elucidated. FOS and XOS can promote the proliferation of *Lactobacillus* in intestine^42,47^. AL inhibits xanthine oxidase activity, preventing the conversion of HX and xanthine to uric acid^3^. We examined the effects of the oral and dietary addition of LGG in the HUA goose model, using FOS, XOS and AL as positive controls. Compared with the HUA group, LGG increased the abundance of the genera *Lactobacillus*, *Butyricicoccus*, and *Ruminococcus* in the gut, decreased the abundance of the genus *Desulfovibrio*, reduced nucleoside transport from the gut to the serum, and hepatic UA anabolism, while enhancing renal UA excretion, and finally systematically alleviated HUA in goose through the gut-liver-kidney axis.

Whole-genome sequencing analysis of LGG revealed that LGG absorbs and degrades nucleosides through three critical genes: nucleoside permease *ABCT*, nucleoside hydrolase *iunH*, and xanthine permease *pbuX*. Heterogeneous expression in *E. coli* and gene knockout in LGG demonstrated that these genes play different roles in nucleoside degradation. Similar to these findings, degradation function genes were also reported in *Lactiplantibacillus plantarum*, which catalyzed the hydrolysis of nucleosides into nucleobases in mice^21^. A recent study also showed that *Clostridium sporogenes* from human subjects has the *pbuX* gene for the conversion of UA to SCFAs^47^.

Natural products from the gut microbial biosynthetic gene cluster represent promising therapeutic agents for animal and human health^48^. Combined with the whole-genome sequencing analysis and metabolomics analysis of extra- and intracellular LGG, we found that the extracellular proline level in LGG was increased under nucleoside pressure by proline excretion genes (*proW*, *proV*). Clinical data showed that proline is negatively correlated with some metabolic diseases, such as diabetes, dyslipidemia, hypertension, HUA, etc^49^. Meanwhile, fecal proline content is significantly reduced in a HUA mouse model^50^. A recent study showed that arginine and proline metabolism are key nodes for distinguishing HUA patients from healthy individuals, and proline alleviates HUA through ascorbate and alternate metabolism^50,51^. Furthermore, proline has been found to protect the kidneys from oxidative stress^51,52^. Our in vitro results in cell lines through the gut-liver-kidney axis also demonstrated that LGG metabolites and proline can decrease intestinal nucleoside transport and UA reabsorption, and down-regulate hepatic purine synthesis, and renal MSU reabsorption. These results shown that there might be a translocation of nucleosides into LGG, therefore extracellular adenosine was reduced and adenosine level in LGG was increased. The cellular results reveal a possible mechanism for the in vivo alleviation of LGG metabolites and proline, that is inhibiting the conversion of adenosine to inosine or UA by suppressing the ADA gene and promoting the expression of the PRODH gene and the ADSS2 gene. However, the consequential relationship between proline metabolism disorders and the occurrence of HUA remains to be verified by further experiments.

Lastly, it is important to note that multi-omics of HUA patients showed gut dysbiosis with the genera *Collinsella* and *Lactobacillus*, consistent with the results in the goose model. The lower abundance of *Lactobacillus* at both the family and genus levels of the HUA population and at the family level in the mouse model indicated that *Lactobacillus* supplementation may be a potential therapy for HUA prevention and alleviation. Proline deficiency and changes in other serum purine-related metabolites in HUA patients showed variations similar to those in the goose model, demonstrating that the serum proline level may be a sensitive index or biomarker for HUA therapy, and proline supplementation could be a viable treatment approach.

In conclusion, our work established a novel model for studying HUA or gout, and we found that LGG plays a role in absorbing and degrading nucleosides, reducing intestinal and renal UA reabsorption, and down-regulating hepatic UA synthesis, which demonstrates its potential as a UA-lowering therapeutic strain. Our results provide new ideas for the alleviation of HUA and its complications and may shed light on possible mechanisms through which *Lactobacillus* can alleviate HUA through the gut-liver-kidney axis.

## Methods

### Ethics statement

This study was approved by the First Affiliated Hospital of Shantou University School of Medicine Ethics Committee (B-2022-155). All participants provided their written informed consent. We recruited 40 male HUA patients (serum UA, 503.4 ± 69.3 μmol/L) and 43 healthy controls (serum UA, 315.9 ± 46.3 μmol/L) for this study. Patients were diagnosed with HUA according to the 2015 ACR/EULAR classification criteria and were shown to be suitable for the treatment in this study^53^. Blood and fecal samples were collected and frozen at −80 °C until analysis.

### Animals

All animal procedures were approved by the Animal Experimentation Ethics Committee of the South China Agricultural University (SYXK-2019-0136). Male 1-day-old healthy goslings were purchased from Guangdong Qingyuan Shixing Biotechnology Co. Ltd., China. Animals were housed in stainless steel cages (four goslings/cage) with free access to water and food and kept in a temperature- controlled room (temperature, maintained at 33 ± 1 °C for the first 3 days and then reduced by 2.5 ± 0.5 °C per week to a final temperature of 26 °C; relative humidity, 45–60%; lighting, 24 h lighting with 10 Lux). Geese were anesthetized by sub-wing intravenous injection and then sacrificed by bloodletting from the jugular vein.

### HUA modeling treatment

A total of 80 1-day-old male Magang geese were randomly divided into two groups, a control (CON) group and a HUA group, with 8 replicates in each group and 5 geese in each replicate. From 1 day to 28 days of age, the CON group was fed a normal diet (CP 16.81%, Ca 1.00%), while the HUA group was fed an HCP diet (CP 24.03% and Ca 3.04%). At 29 days, one goose per replicate, with a weight close to the average, was slaughtered in each group. The formula and nutrient levels for the experimental diets are shown in Table 1.

### Antibiotic treatment

A total of 80 1-day-old male Magang geese were randomly divided into two groups, the HUA group and ANTI group with 8 replicates per group and 5 geese per replicate. From 1 day to 28 days of age, the HUA and ANTI groups were fed the HCP diet (CP 24.03% and Ca 3.04%). The ANTI group was treated with antibiotics (ampicillin 1 g/L + Streptomycin 1 g/L + neomycin 0.5 g/L + chloramphenicol 1 g/L) from 14 days to 28 days of age. At 29 days, one goose per replicate, with a weight close to the average, was anesthetized and sacrificed in each group.

### FMT treatment

A total of 80 1-day-old male Magang geese were selected for the donor group of FMT. The donor group was divided into two treatment groups: a CON group and a HUA group, each group had 8 replicates with 5 geese per replicate. The CON group was fed a normal diet (CP 16.81%, Ca 1.00%), and the HUA group was fed an HCP diet (CP 24.03% and Ca 3.04%). After 14 days of feeding, another 80 1-day-old male Magang geese were selected for the recipient group, and were randomly divided into FMT(CON) and FMT(HUA) groups, which both received a basal diet. For FMT, 10 g fresh fecal samples were collected from the donor groups and resuspended in 50 mL sterile anaerobic saline, vortexed for 3 min, and allowed to settle by gravity for 2 min. Transplant into recipient goslings was achieved by gavage with 10 mL/kg body weight of the supernatant from the fecal sample once a day for 2 weeks. The body weights of the animals were regularly monitored during the treatment period. At 29 days, one goose per replicate, with a weight close to the average, was anesthetized and sacrificed in each group.

### LGG and LGG with metabolite gavage treatment

A total of 256 1-day-old male Magang geese were selected and divided into 2 groups. The CON group was fed a basal diet (CP 16.81%, Ca 1.00%), and the HUA group was fed an HCP diet (CP 24.03%, Ca 3.04%). After successful HUA modeling for 14 days, the HUA group was randomly divided into three groups (B, C, and D). Each group contained 8 replicates with 5 geese per replicate. From 14 to 28 days, groups A and B were gavaged with PBS, group C was gavaged with LGG + PBS, and group D was gavaged with LGG + Metabolites (LGG content > 1 × 10^10^ CFU). At 29 days, one goose per replicate, with a weight close to the average, was anesthetized and sacrificed in each group.

### Probiotics and prebiotic treatment

A total of 360 healthy 1-day-old male Magang geese were randomly divided into 6 groups with 6 replicates per group and 10 geese per replicate according to average body weight. The CON group was fed a normal diet (CP 16.81%, Ca 1.00%) from 1 to 28 days. The five treatment groups were fed the HCP diet (CP 24.03% and Ca 3.04%) from 1 to 28 days. From 14 days of age, the AL group was supplemented with 60 mg/kg AL in the diet, the FOS group was supplemented with 0.4% fructose-oligosaccharide in the diet, the XOS group was supplemented with 0.3% xylooligosaccharides in the diet, and the LGG group was supplemented with 0.1% LGG powder (>1 × 10^10^ CFU/kg) in the diet. At 29 days, one goose per replicate, with a weight close to the average, was anesthetized and sacrificed in each group.

### Intraperitoneal injection in mice

Twenty 5-week-old (25 g) specific pathogen-free Kunming mice were purchased from the Guangdong Provincial Medical Laboratory Animal Center and randomly divided into two groups after 7 days of acclimatization. The CON group was injected intraperitoneally with sodium carboxymethylcellulose and the HUA group was injected intraperitoneally with a solution of potassium oxyzincate and HX formulated from sodium carboxymethylcellulose once daily for 2 weeks in a trial, with free access to water and food. Knee joint thickness was measured by vernier calipers at 21 days. All mice were humanely euthanized with CO_2_ overdose followed by cervical dislocation per the guidelines.

### Sample collection

Following treatment, the animals were sacrificed by jugular vein bloodletting (at 09:00). Individual blood samples were drawn from the jugular vein, and serum samples were separated by centrifuging the blood at 1200 *g* for 10 min at 4 °C, and stored at −30 °C. The complete liver, kidney, duodenum, jejunum, ileum, cecum, and rectum were taken and weighed for conventional analysis. After washing with phosphate-buffered saline (PBS; pH = 7.2–7.4), the central sections of the liver, kidney, and jejunum samples (~1–2 cm) were taken and fixed in fresh 4% paraformaldehyde for paraffin embedding. Parts of the liver, kidney, and jejunum mucosa were flash-frozen in liquid nitrogen for mRNA and protein extraction for qRT-PCR and WB. Cecum chyme was collected and refrigerated at −80 °C. Throughout the treatment period, the animals’ body weights were checked periodically.

### Analysis of biochemical parameters

Serum biochemical indexes, including UA, XOD, urea nitrogen, creatinine, calcium, and phosphorus, were detected by an automatic biochemical analyzer. The contents of inflammatory cytokines interleukin-1beta (IL-1β), tumor necrosis factor-alpha (TNF-α), and interferon-gamma (IFN-γ) in liver tissue and serum were determined according to the instructions provided by the manufacturer of an Enzyme-Linked Immunosorbent assay kit (Nanjing Jiancheng Institute of Bioengineering, China).

### Western blot analysis

The samples were lysed in RIPA (Sigma‐Aldrich) buffer supplemented with protease and phosphatase inhibitors. The lysates were then diluted to a concentration of 20 μg of protein and heated at 95 °C for 5 min with a denaturation buffer. The proteins were separated by SDS-PAGE electrophoresis and transferred to PVDF membranes (Amersham International, GE Healthcare). Membranes were incubated in blocking reagent (3% Amersham ECL Prime Blocking solution reagent) in Tris-buffered saline-Tween 20 (TBS-Tween) for 1 h, then overnight in primary antibody (in the blocking solution) at 4 °C. The antibodies and their concentrations were as follows: anti-CNT2 (1:500, DF4522, Affinity Biosciences), anti-TJP1 (1:2000, 21773-1-AP, Proteintech), anti-URATI (1:1000, bs-10357R, Bioss), anti-GLUT9 (1:1000, 26486-1-AP, Proteintech), anti-ABCG2 (1:1000, ab108312, Abcam), anti-OAT1 (1:1000, ab135924, Abcam), anti-PPAT (1:1000, 15401-1-AP, proteintech), anti-PRPS (1:1000, bs-4504R, Bioss), anti-ADA (1:1000, 13328-1-AP, Proteintech), anti-XOD (1:1000, ab109235, Abcam), and anti-β-actin (1:5000, 60009-1-Ig, Proteintech). After multiple washes in TBS-Tween, the membranes were incubated in horseradish peroxidase (HRP)-conjugated secondary antibodies for 1 h at room temperature in the blocking solution. Subsequently, the membranes were treated with ECL western blotting substrate (Amersham International, GE Healthcare, Chicago, IL, USA) and imaged using a chemiluminescence detection system (Bio‐Rad Laboratories, Hercules, CA, USA). The band intensity was quantified using ImageJ software. All antibiotics are listed in Supplementary Data 1.

### Real‐Time PCR

Total RNA from goose tissues, cell lines, and strains was extracted using Trizol (Invitrogen, Waltham, MA, USA) according to the manufacturer’s instructions. Next, messenger RNAs were reverse transcribed to cDNAs using the Color Reverse Transcription Kit (EZBioscience, Shanghai, China). A quantitative real‐time polymerase chain reaction (qRT-PCR) was performed to measure gene expression. The cycle threshold values obtained from samples were compared using the 2^–ΔCt^ method. For the detection of goose and cell line genes, β-actin served as the internal reference gene. For the detection of LGG genes, 16S rRNA served as the reference gene. All primers are listed in Supplementary Table 1.

### Untargeted/Targeted metabolomics analysis

Metabolites were extracted with 500 μL of extraction solution (methanol: acetonitrile: water = 2:2:1 (V/V), containing isotope-labeled internal standard mixture), and the supernatant was used for LC-MS/MS analysis. LC-MS/MS analyses were conducted using a UHPLC system (Vanquish, Thermo Fisher Scientific, Waltham, MA, USA) with a UPLC BEH Amide column (2.1 mm × 100 mm, 1.7 μm) connected to a Q Exactive HFX mass spectrometer (Orbitrap MS, Thermo Fisher). The mobile phase consisted of 25 mmol/L ammonium acetate and 25 ammonia hydroxides in water, with a pH of 9.75. The autosampler was set at a temperature of 4 °C, and the injection volume was 2 μL. For the acquisition of MS/MS spectra, the QE HFX mass spectrometer was used in information-dependent acquisition mode, controlled by the Xcalibur software from Thermo Fisher. In this mode, the software consistently assesses the full-scan MS spectrum. The raw data were converted to the mzXML format using ProteoWizard. Subsequently, an in-house program, developed with R and based on XCMS, was used for peak detection, extraction, alignment, and integration. Metabolite annotation was performed using an in-house MS2 database called BiotreeDB. The annotation cutoff was set at 0.3.

### Metagenomic sequencing

Total genomic DNA was extracted from cecal chyme samples using the E.Z.N.A.® Soil DNA Kit (Omega Bio-Tek, Norcross, GA, U.S.) following the manufacturer’s instructions. The concentration and purity of the extracted DNA were assessed using TBS-380 and NanoDrop2000, respectively. The quality of the DNA extract was evaluated on 1% agarose gel. DNA extract was fragmented to an average size of approximately 400 bp using a Covaris M220 device (Gene Company Limited, China) to construct a paired-end library. The construction of the paired-end library was carried out using the NEXTflexTM Rapid DNA-Seq kit (Bioo Scientific, Austin, TX, USA). Adapters with complete sequencing primer hybridization sites were ligated to the blunt end of fragments. Paired-end sequencing was conducted on an Illumina Hiseq Xten device (Illumina Inc., San Diego, CA, USA) at Majorbio Bio-Pharm Technology Co., Ltd. (Shanghai, China) using HiSeq X Reagent Kits, following the manufacturer’s instructions (www.illumina.com). Sequence data associated with this project have been deposited in the NCBI Short Read Archive database (Accession Number: PRJNA1068249).

### 16S rRNA sequencing

Microbial community genomic DNA was extracted from cecal stool using the E.Z.N.A.® soil DNA Kit (Omega Bio-Tek, Norcross, GA, USA) following the manufacturer’s instructions. The DNA extract was analyzed on 1% agarose gel, and the DNA concentration and purity were measured using a NanoDrop 2000 UV-vis spectrophotometer (Thermo Fisher Scientific, Wilmington, NC, USA). The V3-V4 hypervariable region of the bacterial 16S rRNA gene was amplified using primer pairs 338F (5′-ACTCCTACGGGAGGCAGCAG-3′) and 806R (5′-GGACTACHVGGGTWTCTAAT-3′) with an ABI GeneAmp® 9700 PCR thermocycler (ABI, CA, USA). PCR amplification of the 16S rRNA gene was carried out using the following protocol: initial denaturation at 95 °C for 3 min, followed by 27 cycles of denaturation at 95 °C for 30 s, annealing at 55 °C for 30 s, and extension at 72 °C for 45 s, a single extension at 72 °C for 10 min, and ending at 4 °C. The PCR product was extracted from 2% agarose gel and purified using the AxyPrep DNA Gel Extraction Kit (Axygen Biosciences, Union City, CA, USA) following the manufacturer’s instructions. Quantification was performed using the Quantus™ Fluorometer (Promega, Madison, WI, USA). Equimolar purified amplicons were paired-end sequenced on an Illumina MiSeq PE300 platform (Illumina, San Diego, CA, USA) following standard protocols by Majorbio Bio-Pharm Technology Co. Ltd. (Shanghai, China). The raw reads were submitted to the NCBI Sequence Read Archive (SRA) database (Accession Number: PRJNA1068249). To ensure the quality of the raw sequencing sequences, fastp software (version 0.20.0) from OpenGene (https://github.com/OpenGene/fastp) was used for quality control. For splicing, FLASH software (version 1.2.7) from the University of Maryland (http://www.cbcb.umd.edu/software/flash) was used. UPARSE software (version 7.1) (http://drive5.com/uparse/) was used to cluster the sequences into operational taxonomic units (OTUs) based on a 97% similarity threshold, while also eliminating chimeras. The RDP classifier (http://rdp.cme.msu.edu/, version 2.2) was used to annotate species classifications on each sequence. The results were compared to the Silva 16S rRNA database (version 138) and the alignment threshold was set to 70%.

### Strains and medium

The strain LGG and the standard strain *E. coli* are maintained by the Waterfowl Nutrition Laboratory of South China Agricultural University. Commercial LB and MRS medium were purchased from Guangdong Huankai Microorganism Technology Co., Ltd.

### Determination of the strain degradation rate

The standard strain *E. coli* was added to the LB liquid medium, while the LGG freeze-dried strain powder was added to the MRS liquid medium. Cultures were then microaerophilically cultured at 37 °C for 12 h. For activation, an inoculum volume of 5% (v/v) was added and continuously activated for three generations. Subsequently, 10% of the inoculum (v/v) was transferred to LB and MRS medium for subsequent experiments. The inosine buffer solution was prepared by dissolving 33.7 mg inosine (1.25 mM) in 100 mL K_3_PO_4_ solution (10 mmol/L, adjusted to pH = 7.0 with H_3_PO_4_). To 0.9 mL of standard solution was added 0.1 mL of reaction terminator (0.1 mol/L HClO_4_), and the mixture was shaken. 20 μL was aspirated for injection analysis. The standard curve (1.25, 0.75, 0.5, 0.25, 0.125 mM; concentration-peak area) was determined based on the external standard method. Afterward, 2 mL of 20 h bacterial liquid medium was obtained, with 4 replicates each, and centrifuged at 6000 r/min at 4 °C for 10 min. After being washed twice with 1 mL of sterile PBS, the solution was resuspended with 750 μL of inosine solution and incubated at 37 °C for 60, 180, 360, and 720 min. The solution was then centrifuged at 4000 *g* for 10 min at 4 °C. To 720 μL of the supernatant was added 80 μL (9:1) HClO_4_ (0.1 mol/L), and the result was mixed to prevent further degradation. After filtration through a 0.22 μm membrane, 20 μL of the mixture was injected into a high-performance liquid chromatography device. HPLC a spursil EP C18 (250 mm × 4.6 mm, 5 μm) reversed-phase column, the mobile phase was 20 mmol/L potassium dihydrogen phosphate solution (pH = 3.0) and 1% methanol, and the flow rate was 1 mL/min. The column temperature was 25 °C, the measurement wavelength was 254 nm, and the elution time was 40 min.

### Heterologous expression and gene knockout

The oligonucleotide primers used in this study were synthesized by the Qingdao branch of Beijing DynaScience Biotechnology Company Limited, and sequencing services were provided by Shenzhen Huada Gene Co. Heterologous expression plasmid p15A-cm-P_genta_-iunH is based on a p15A origin and harbors the *iunH* gene under the P_genta_ promoter. A fragment containing p15A-cm was amplified from p15A-cm-hyg-ccdB, a fragment containing the *iunH* gene was amplified by colony PCR, and a fragment containing the P_genta_ promoter was amplified from r6k-loxM-genta. These three fragments were co-transformed into GB05-dir induced for the expression of RecET, followed by selection on LB plates containing 15 μg/mL chloramphenicol for linear plus linear homologous recombination to generate p15A-cm-P_genta_-iunH^54^. Construction of other heterologous expression plasmids was the same as that for p15A-cm-P_genta_-iunH except that different homologous expression genes. The gene knockout plasmid p15A-cm-HA-G-iunH-erm-sacB is based on a p15A origin and harbors 1 kb HA on each side of the *iunH*. For the initial construct, a fragment containing p15A-cm was amplified from p15A-cm-hyg-ccdB, a fragment containing the erm resistance gene was amplified from pBBR1-Rha-redγ-Kan-erm, two fragments containing the homologous arms (HAR and HAL) were amplified by colony PCR and a fragment containing the sacB gene was amplified from rk2-apra-sacB. These five fragments were co-transformed into GB05-dir induced for the expression of RecET, followed by selection on LB plates containing 15 μg/mL chloramphenicol for linear plus linear homologous recombination to generate p15A-cm-HA-G-iunH-erm-sacB. Construction of other gene knockout plasmids was the same as that for p15A-cm-HA-G-iunH-erm-sacB except that different homologous arms were used. The recombinant strains were screened for resistance and single colonies were selected for PCR validation to obtain successful recombinant deletion strains. All strains, plasmids, mutants, and primers used in this study are listed in Supplementary Table 2.

### IPEC-J2 cell experiments

IPEC-J2 cells were seeded in 96-well plates with 2 × 10^4^ cells per well in DMEM medium. There were 6 replicates per group (*n* = 6). Treatments were performed with 0, 30, 100, 300, 500, 1000, and 3000 μM nucleosides (inosine) for 2, 4, 6, 8, 10 and 12 h. Absorbance was measured using a CCK8 reagent and cell viability was calculated. LGG was cultured in MRS medium to the logarithmic growth phase, and the supernatant was collected by centrifugation at 4000 *g* for 5 min and filtered at 0.22 μm. LGG supernatant metabolites were added to the cell culture medium at 0, 5%, 10%, 25%, and 50%. Cells were treated with proline in a concentration gradient of 0, 10, 50, 100, 500, and 1000 μM. Six parallels of each concentration were treated for 12 h and 24 h, respectively, and the absorbance was measured by CCK-8 reagent to calculate cell viability. According to the CCK8 results, inosine, LGG supernatant metabolites, and proline were selected to treat cells at a certain concentration and treatment time. The upper layer of the medium was discarded, cells were rinsed 1–2 times with PBS, and total RNA was extracted to determine the expression of CNT2, TJP1, ABCG2, and GLUT9 genes.

### Hep-G2 cell experiments

Hep-G2 cells were seeded in 96-well plates with 2 × 10^4^ cells per well in DMEM medium. There were 6 replicates per group (*n* = 6). Treatments were performed with 10, 50, 100, 200, 300, 400, and 500 μM HX for 2, 4, 6, 8, 10 and 12 h. Absorbance was measured using a CCK8 reagent and cell viability was calculated. LGG was cultured in MRS medium to the logarithmic growth phase, and the supernatant was collected by centrifugation at 4000 *g* for 5 min and filtered at 0.22 μm. LGG supernatant metabolites were added to the cell culture medium at 0, 5%, 10%, 25%, and 50%. Cells were treated with proline in a concentration gradient of 0, 10, 100, and 350 μM. Six parallels of each concentration were treated for 12 h and 24 h, respectively, and the absorbance was measured by CCK-8 reagent to calculate cell viability. According to the CCK8 results, HX, LGG supernatant metabolites, and proline were selected to treat cells at selected concentrations and treatment times. The upper layer of the medium was discarded, cells were rinsed 1–2 times with PBS, and total RNA was extracted to determine the expression of CNT2, TJP1, ABCG2, and GLUT9 genes.

### BHK cell experiments

NaOH solution was added to pure water as the previous study described previously, and uric acid was added after boiling and stirring to dissolve. The solution was adjusted to pH 8.9 and then refrigerated at 4 °C overnight. The next day, the supernatant was discarded by suction filtration, and the sample was dried to obtain MSU crystal powder. MSU crystal suspension was prepared with sterile normal saline. BHK cells were seeded in 96-well plates with 2 × 10^4^ cells per well in DMEM medium. There were 6 replicates per group (*n* = 6). Treatments were performed with 0, 20, 50, 75, 100, 150, and 200 μM MSU for 2, 4, 6, 8, 10 and 12 h. Absorbance was measured using a CCK8 reagent and cell viability was calculated. Cells were treated with a proline concentration gradient of 0, 50, 250, 500, and 1000 μM, with 6 replicates for each concentration, respectively, for 2, 4, 6, 8, 10, and 12 h, and the absorbance was measured using CCK-8 reagent to calculate cell viability. According to the CCK8 results, MSU, LGG supernatant metabolites, and proline were selected to treat cells at three concentrations and one treatment time, respectively. The upper layer of the medium was discarded, cells were rinsed 1–2 times with PBS, and total RNA was extracted to determine the expression of CNT2, TJP1, ABCG2, and GLUT9 genes.

### RNA Fluorescence in situ Hybridization (FISH) Assay

We first designed and obtained a FAM-labeled LGG genus-specific FISH probe from GENERAL BIOL (Anhui, China) with the sequence ATATTCCTGTTCTCCGCGGTTCTGCC. A FISH kit (GenePharma, China) was used to detect the signal of the probe according to the manufacturer’s protocol. Images were acquired by an inverted fluorescence microscope system (ECLIPSE Ci-L, Nikon).

### Statistical analysis

Values are expressed as means ± SEM. Statistical analysis was performed using the SAS 9.2 (SAS Inst. Inc., Cary, NC, USA). Significant differences between the two groups were evaluated by two-tailed unpaired Student’s *t*-test or a Mann-Whitney *U* test for samples that were not normally distributed. Significant differences among three or more groups were evaluated by one-way ANOVA with Bonferroni’s multiple comparisons test. *, *p* < 0.05; **, *p* < 0.01; ***, *p* < 0.001; ****, *p* < 0.0001; ns no significance.

### Reporting summary

Further information on research design is available in the Nature Research Reporting Summary linked to this article.

### Supplementary information

Supplementary Information

Reporting Summary

## Data Availability

Datasets of the 16S rRNA and metagenome are available in the National Center for Biotechnology Information under BioProject accession numbers PRJNA1068249. The de novo *Lactobacillus rhamnosus* GG genome sequences are available under BioProject accession number PRJNA1068249. Raw data for serum metabolomics are presented in figshare, and doi are 10.6084/m9.figshare.25097426, 10.6084/m9.figshare.25048334, 10.6084/m9.figshare.25097408, and 10.6084/m9.figshare.25097402. Raw data for figures are presented in figshare, and doi is 10.6084/m9.figshare.25097378. The data that support the findings of this study are available from the corresponding author upon request.
