# Supplementary material for: Lactobacillus rhamnosus GG ameliorates hyperuricemia in a novel model
Source: NPJ Biofilms Microbiomes. 2024 Mar 20;10:25. doi: 10.1038/s41522-024-00486-9 (PMC10954633; doi:10.1038/s41522-024-00486-9)

**
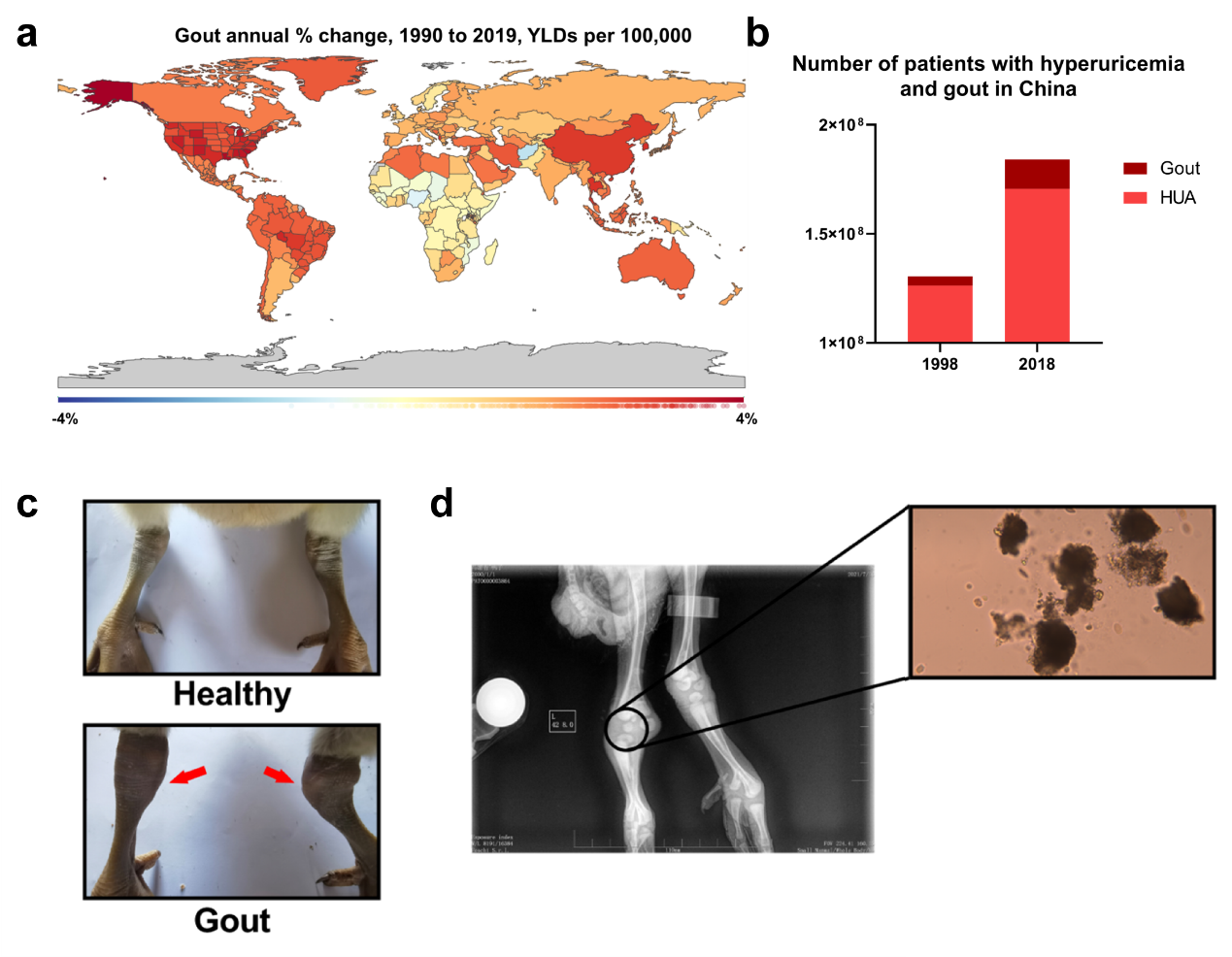
Supplementary Figure 1. Geese have potential as a model animal for the study of hyperuricemia and gout.**

**a**, Global changes of percentage of gout from 1990 to 2019. YLDs: Years lived with disability. The data in the figure refer to the results of the Global Burden of Disease survey. **b**, Number of people with gout and hyperuricemia in China. The data in the figure refer to guidelines for the diagnosis and treatment of Hyperuricemia and Gout in China. **c**, Swollen joints of geese suffering from gout. **d**, Deposition of uric acid crystals in the joints of gout geese.

**
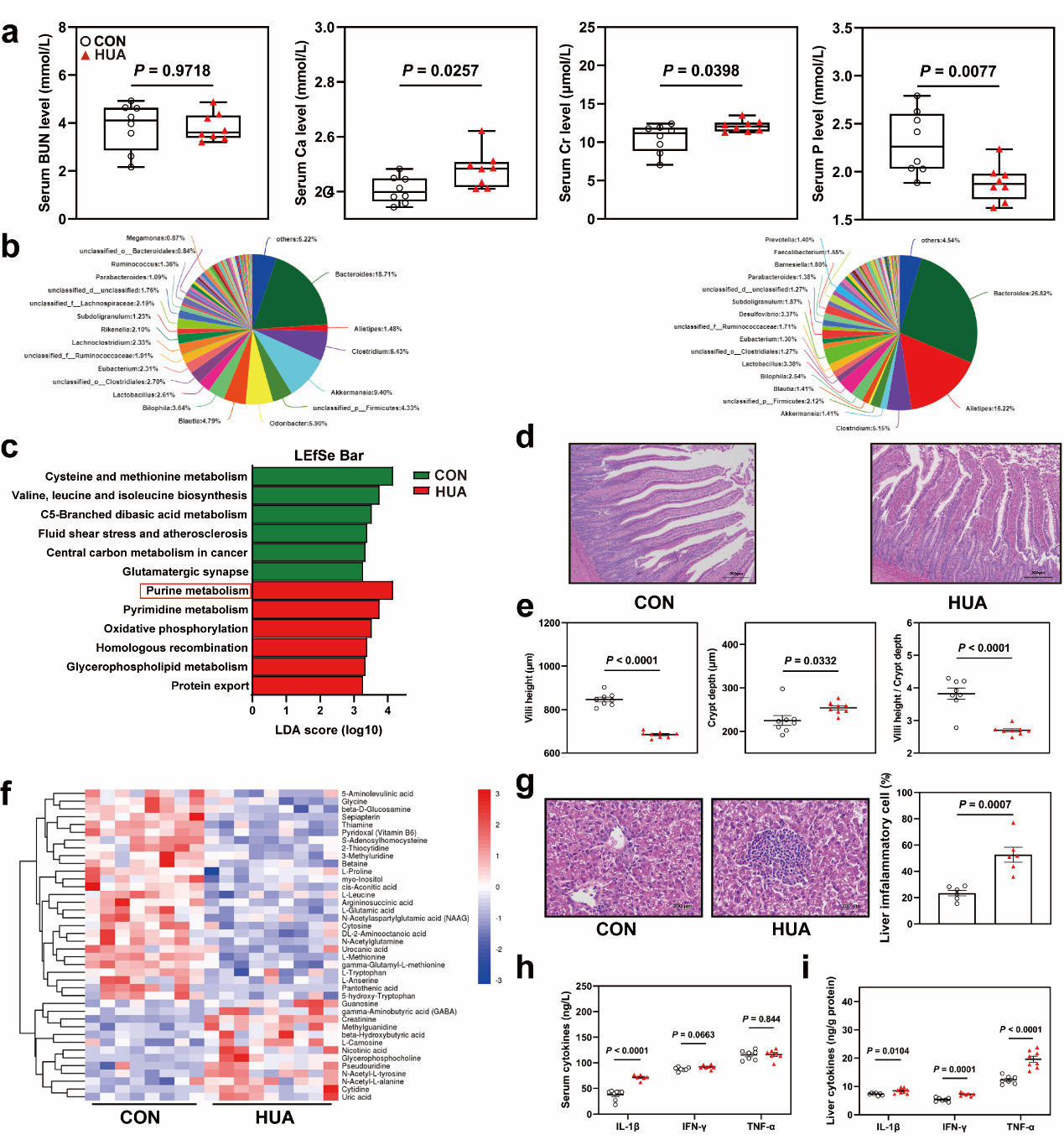
Supplementary Figure 2. HCP diet constructs a gosling HUA model.**

**a**, Effect of HCP diet on the serum creatinine (Cr), blood urea nitrogen (BUN), serum calcium (Ca) and phosphorus (P) levels (n = 8). **b**, Relative abundance of bacteria at the genus level with CON group (left, n = 7) and HUA group (right, n = 7). **c**, LDA scores in KEGG enrichment pathways analysis between CON group (green, n = 7) and HUA group (red, n = 7). **d**, Representative images of H&E staining in jejunum sections from CON group or HUA group (×400, n = 8). All scale bars are 200 μm. **e**, Villi height, crypt depth, and the value of villi height/crypt depth (n = 8). 8 crypts were counted for each section. **f**, Heatmap of LC-MS data between CON group and HUA group (n = 8). Increases in metabolite levels are shown in red, whereas blue indicates decreased metabolite. **g**, Representative images of H&E staining in liver sections from CON group or HUA group (×200, n = 8). All scale bars are 100 μm. **h**, Effect of HCP diet on the levels of inflammatory cytokines (IL-1β, IFN-γ, and TNF-α) in the liver tissue between CON group and HUA group (n = 8). **i**, Effect of HCP diet on the levels of inflammatory cytokines (IL-1β, IFN-γ, and TNF-α) in the serum between CON group and HUA group (n = 8). Data with error bars represent mean ± s.e.m. For a, f, h, i, data were analysed by two-tailed unpaired Student’s t test.

**
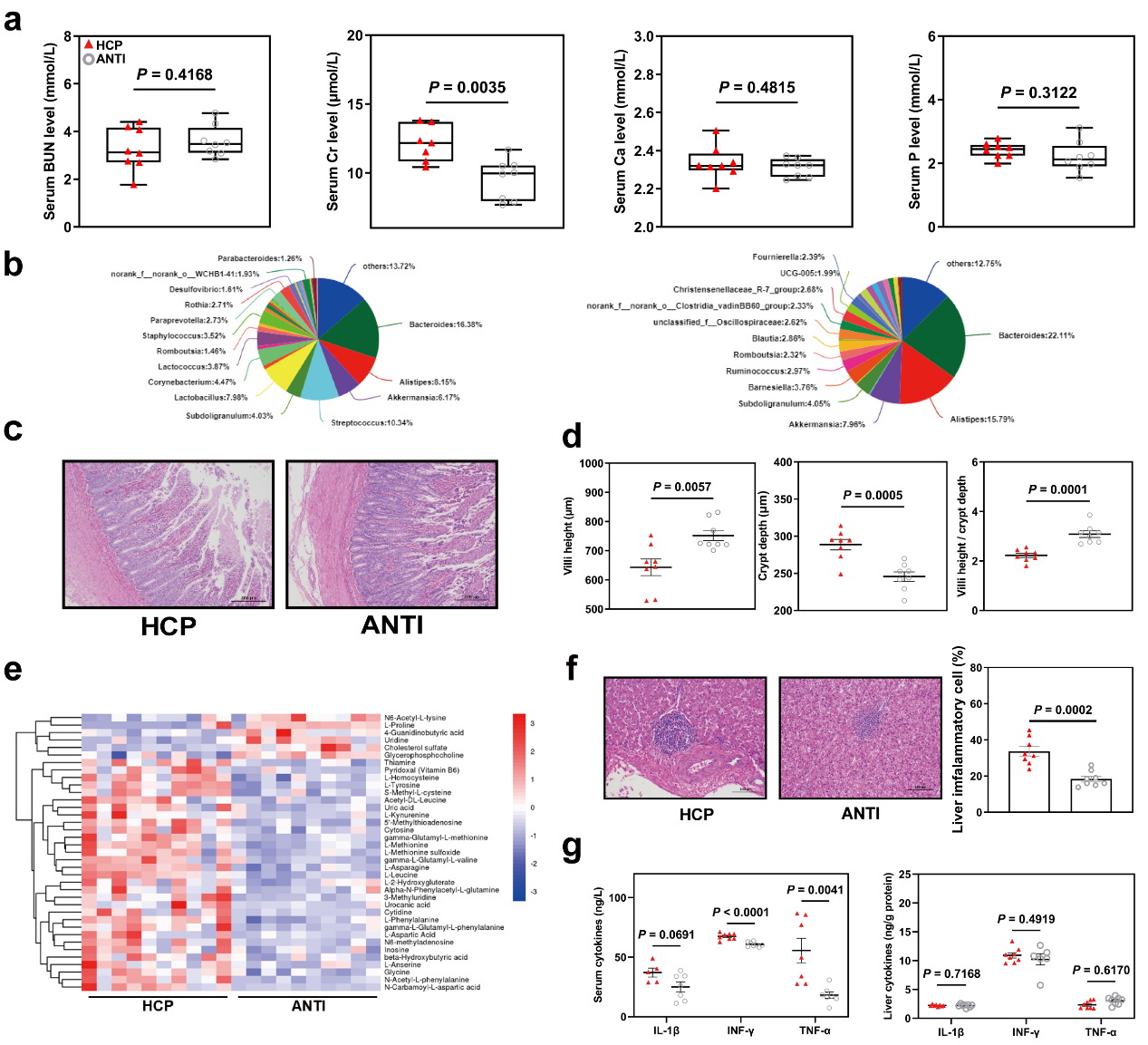
Supplementary Figure 3.** **Antibiotics treatment alleviates HCP diet-induced HUA.**

**a**, Effect of HCP diet on the serum creatinine (Cr), blood urea nitrogen (BUN), serum calcium (Ca) and phosphorus (P) levels (n = 8). **b**, Relative abundance of bacteria at the genus level with HUA group (above, n = 6) and ANTI group (below, n = 7). **c**, Representative images of H&E staining in jejunum sections from HUA group and ANTI group (×400, n = 8). All scale bars are 200 μm (above). **d**, Effect of antibiotics treatment on Villi height, crypt depth, and the value of villi height/crypt depth in HCP diet-treated geese (below, n = 8). 8 crypts were counted for each section. **e**, Heatmap of serum LC-MS data between HUA group and ANTI group (n = 8). Increases in metabolite levels are shown in red, whereas blue indicates decreased metabolite. **f**, Representative images of H&E staining in liver sections from HUA group and ANTI group (×200, n = 8). All scale bars are 100 μm. **g**, Alterations of Levels of inflammatory cytokines (IL-1β, IFN-γ, and TNF-α) in the liver tissue or serum between HUA group and ANTI group (n = 8). Data with error bars represent mean ± s.e.m. For a, c, and f, data were analysed by two-tailed unpaired Student’s t test.

**
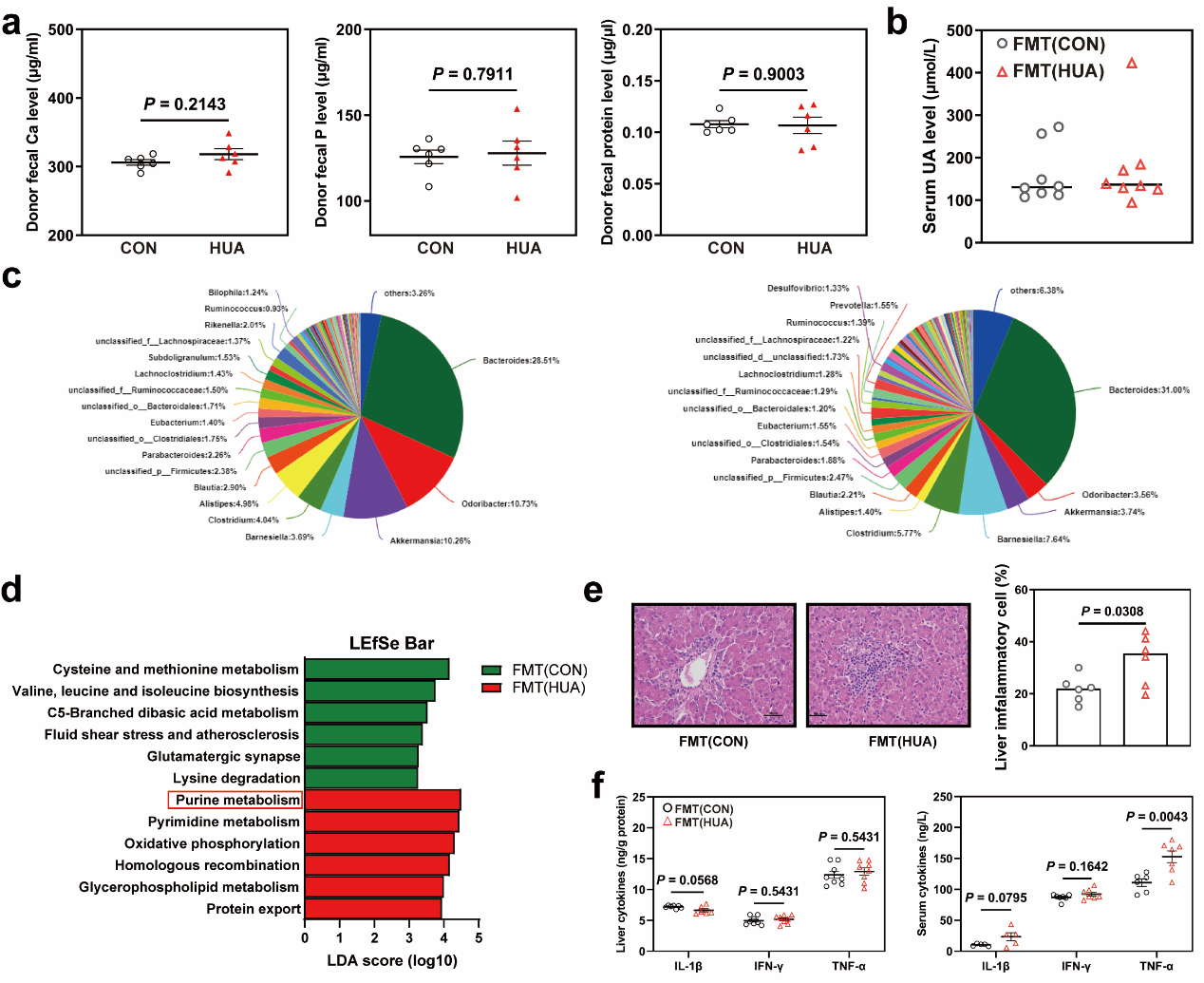
Supplementary Figure 4. FMT disordered bacteria group and induced the occurrence of HUA through gut-liver-kidney axis.**

**a**, Protein, Ca, and P levels in the feces of the donor group (n = 6). Protein was determined by BCA kit. Ca，P was determined by flame atomic absorption spectrophotometry. **b**, Effect of FMT on the serum UA levels (n = 8). **c**, Relative abundance of bacteria at the genus level with FMT(CON) group (above, n = 6) and FMT(HCP) group (below, n = 6). **d**, LDA scores in KEGG enrichment pathways analysis between FMT(CON) group (green, n = 6) and FMT(HCP) group (red, n = 6). **e**, Representative images of H&E staining in liver sections from FMT(CON) group and FMT(HCP) group (×400, n = 6). All scale bars are 50 μm. **f**, Alterations of levels of inflammatory cytokines (IL-1β, IFN-γ, and TNF-α) in the liver tissue and serum between FMT(CON) group and FMT(HCP) group (n = 8, mean with SEM). Data with error bars represent mean ± s.e.m. For a, e, and f, data were analysed by two-tailed unpaired Student’s t test.

**
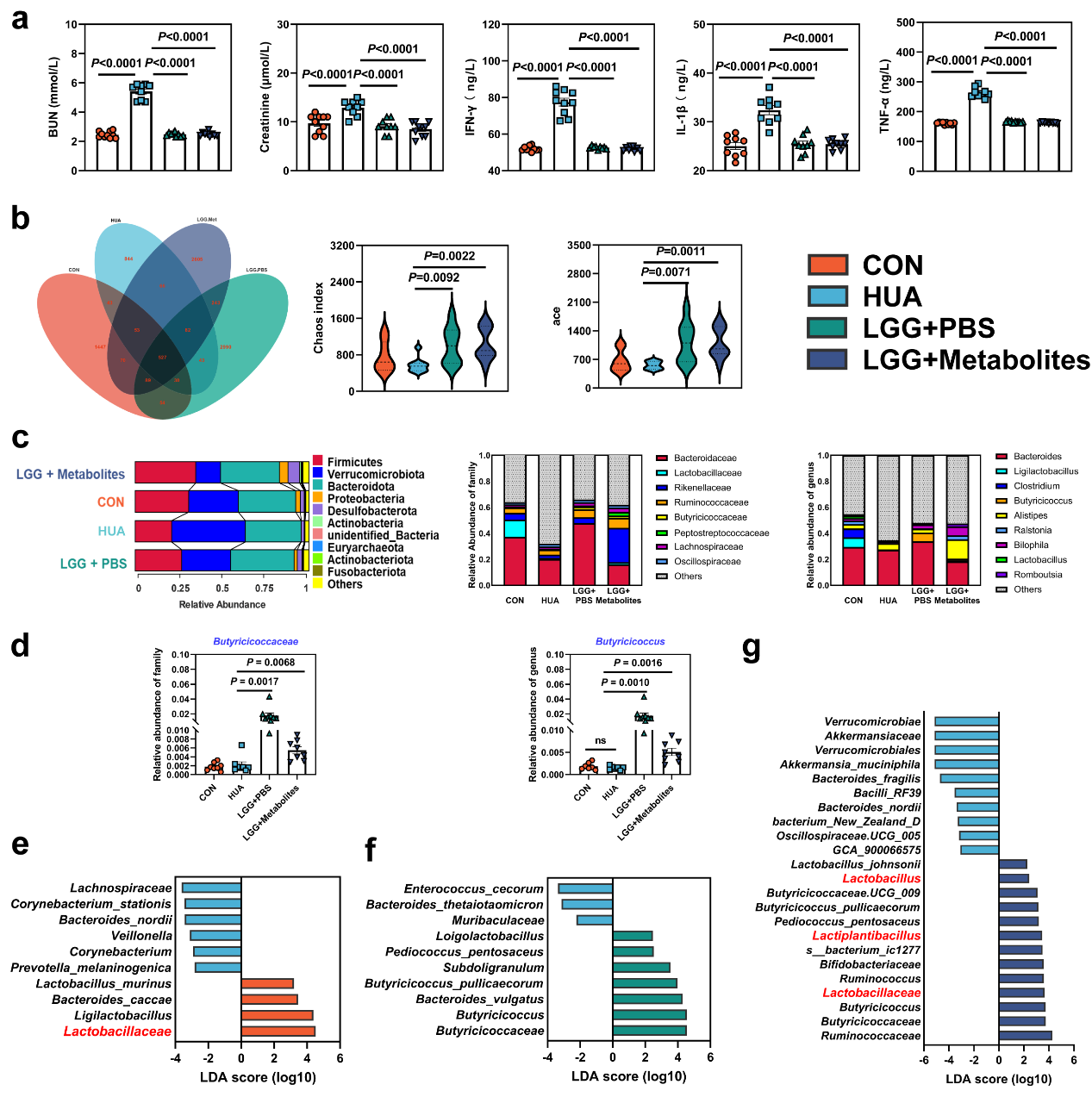
Supplementary Figure 5.** **LGG and LGG metabolites treatment alleviates HCP diet-induced HUA.**

**a**, Effect of LGG and LGG metabolites treatment on the serum creatinine (Cr), blood urea nitrogen (BUN), and inflammatory cytokines (IL-1β, IFN-γ, and TNF-α) levels (n = 8). **b**, Flora OTU abundance of indicated groups (left). Chao and Ace index of indicated groups based on alpha diversity analysis (n = 8). **c**, The alteration trends of the bacterial relative abundance (n = 8). **d**, Butyricicoccus and Butyricicoccaceae relative abundance between HUA group and CON group, LGG+PBS group, LGG+Metabolites group (n = 8). **e**, LDA scores in differential flora enrichment analysis between HUA group and CON group. **f**, LDA scores in differential flora enrichment analysis between HUA group and LGG+PBS group. **g**, LDA scores in differential flora enrichment analysis between HUA group and LGG+Metabolites group. Data with error bars represent mean ± s.e.m. For a, b, and d, data were analysed by two-tailed unpaired Student’s t test. LGG+PBS: LGG cells resuspended in PBS., LGG+Metabolites: LGG cells and its metabolites.

**
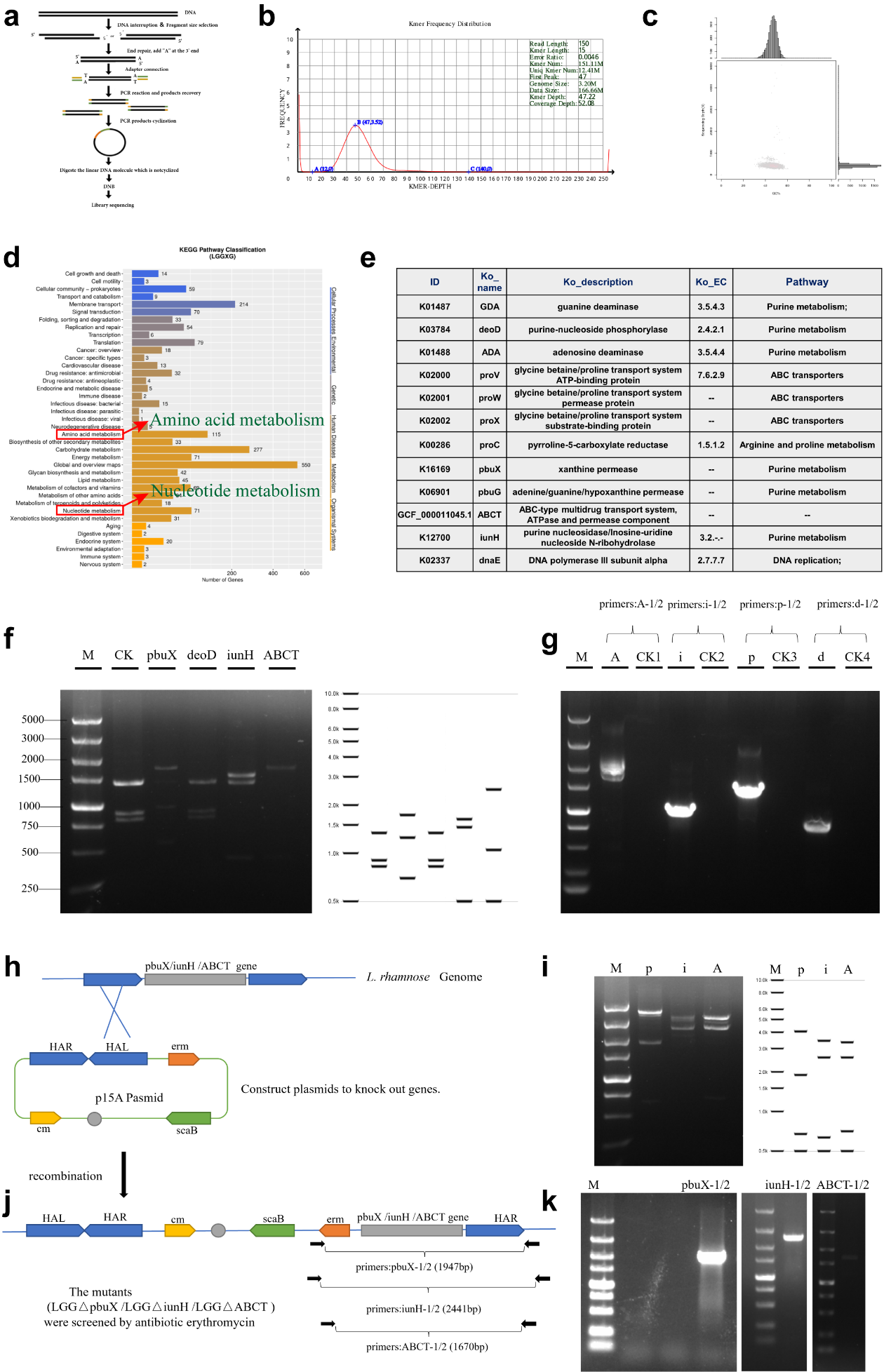
Supplementary Figure 6.** **Whole genome sequence, heterologous expression and knock out genes of LGG.**

**a**, Pipeline of Experiment. **b**, 15-mer analysis on sample. **c**, GC content and Depth correlative analysis. **d**, Gene enrichment analysis of the KEGG pathway. **e**, Purine nucleoside metabolism and proline metabolism pathway gene retrieval. **f**, p15A cm Pgenta; p15A cm-LGGpbuX; p15A cm-LGGdeoD; p15A cm LGGiunH; p15A cm LGGABCT restriction Endonuclease digestion map. M: DL 5000 DNA marker, with stripe sizes ranging from top to bottom from 5 kb, 3 kb, 2 kb, 1.5 kb, 1 kb, 0.75 kb, 0.5 kb, 0.25 kb, and 0.1 kb. **g**, PCR verification of the heterologous expression of recombinant plasmids p15A cm-Pgenta, p15A-cm-LGGiunH, p15A-cm-LGGABCT, p15A-cm-LGGdeoD, p15A-cm-LGGpbuX. A1: Pgenta-LGABCT-1, A2: LGABCT-2, i1: Pgenta-LGiunH-1, i2: LGiunH-2, p1: Pgenta-LGpbuX-1, p2: LGpbuX-2, d1: Pgenta-LGpbuX-1, d2: LGdeoD, right: size of PCR product: 1781bp (mutants, A1/A2), 1030bp (mutants, i1/i2), 1425bp (mutants, p1/p2), 763bp (mutants, d1/d2), M: DL5000 DNA ladder, CK: Nissle1917/p15A cm Pgenta. **h,** Construct plasmids to knock out genes. **i,** p15A cm-HA-erm scaB GpbuX, p15A cm-HA-erm scaB GiunH, and p15A cm-HA-erm scaB GABCT restriction Endonuclease digestion map. M: DL 5000 DNA marker. **j,** The recombinant strains were screened for resistance and single colonies were selected for PCR validation to obtain successful recombinant deletion strains. **k,** PCR verification of the heterologous expression of recombinant plasmids p15A cm-HA-erm scaB GpbuX, p15A cm-HA-erm scaB GiunH, and p15A cm-HA-erm scaB GABCT, pbuX-1: check-LGpbuX-1, pbuX-2: check-LGpbuX-2, iunH-1:check-LGiunH-1, iunH-2: check-LGiunH-2, ABCT-1: check-LGABCT-1, ABCT-2: check-LGABCT-2, Right: size of PCR product: 1947bp (mutants, pbuX-1/2), 2441bp (mutants, iunH-1/2), 1670bp (mutants, ABCT-1/2), M: DL5000 DNA ladder.

**
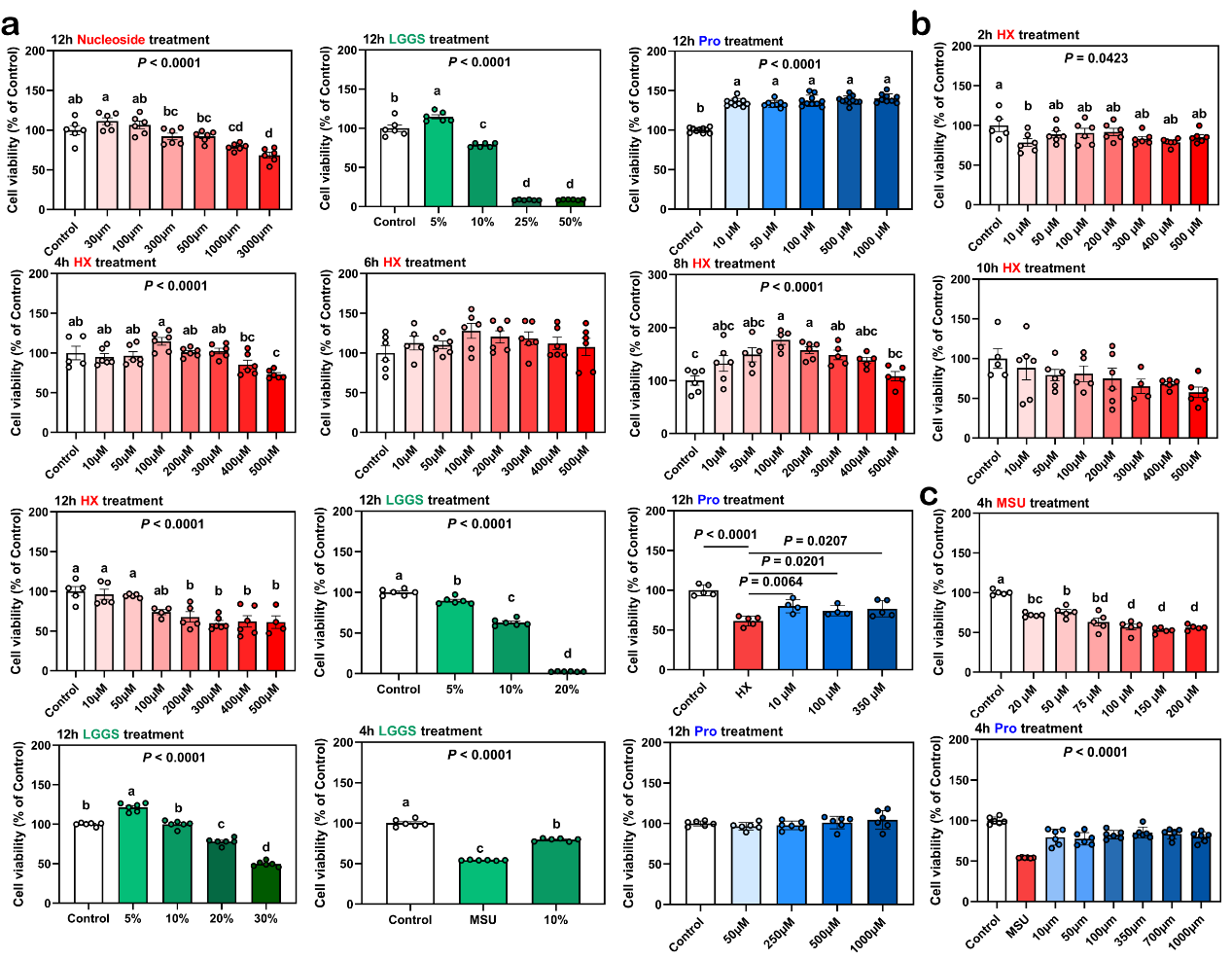
Supplementary Figure 7.** **CCK-8 determines treatment time and concentration.**

**a**, Effects of nucleosides, LGG metabolites or proline on intestinal IPEC-J2 cell viability (n = 8). **b**, Effects of HX, LGG metabolites or proline on liver Hep-G2 cell viability (n = 6). **c**, Effects of MSU, LGG metabolites or proline on liver Hep-G2 cell viability (n = 6). Data with error bars represent mean ± s.e.m. For a, c, and d, data were employed for One-way ANOVA statistical analysis. LGGS: LGG metabolites solution, HX: hypoxanthine, MSU: Sodium urate.

**
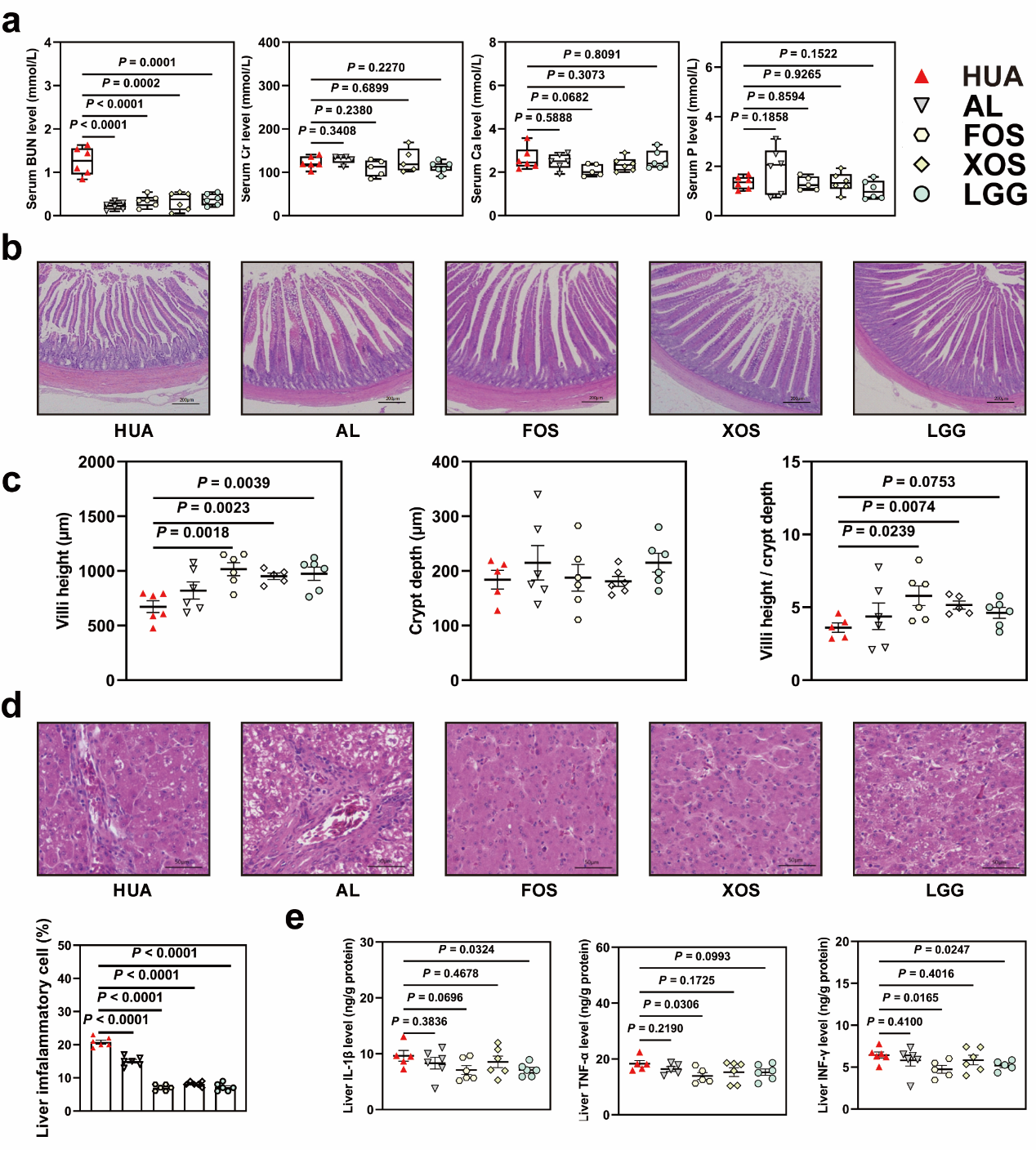
Supplementary Figure 8.** **LGG treatment alleviates HCP diet-induced HUA.**

**a**, Effect of LGG, AL and prebiotics on the serum creatinine (Cr), blood urea nitrogen (BUN), serum calcium (Ca) and phosphorus (P) levels (n = 6). **b**, Representative images of H&E staining in jejunum sections indicated groups (×400, n = 6). All scale bars are 200 μm. **c**, Villi height, crypt depth, and the value of villi height/crypt depth (n = 6). 8 crypts were counted for each section. **d**, Representative images of H&E staining in liver sections from indicated groups (×400, n = 6). All scale bars are 50 μm. **e**, Levels of inflammatory cytokines (IL-1β, IFN-γ, and TNF-α) in the liver tissue of indicated groups (n = 6). Data with error bars represent mean ± s.e.m. For a, c, and e, data were analysed by two-tailed unpaired Student’s t test.

**
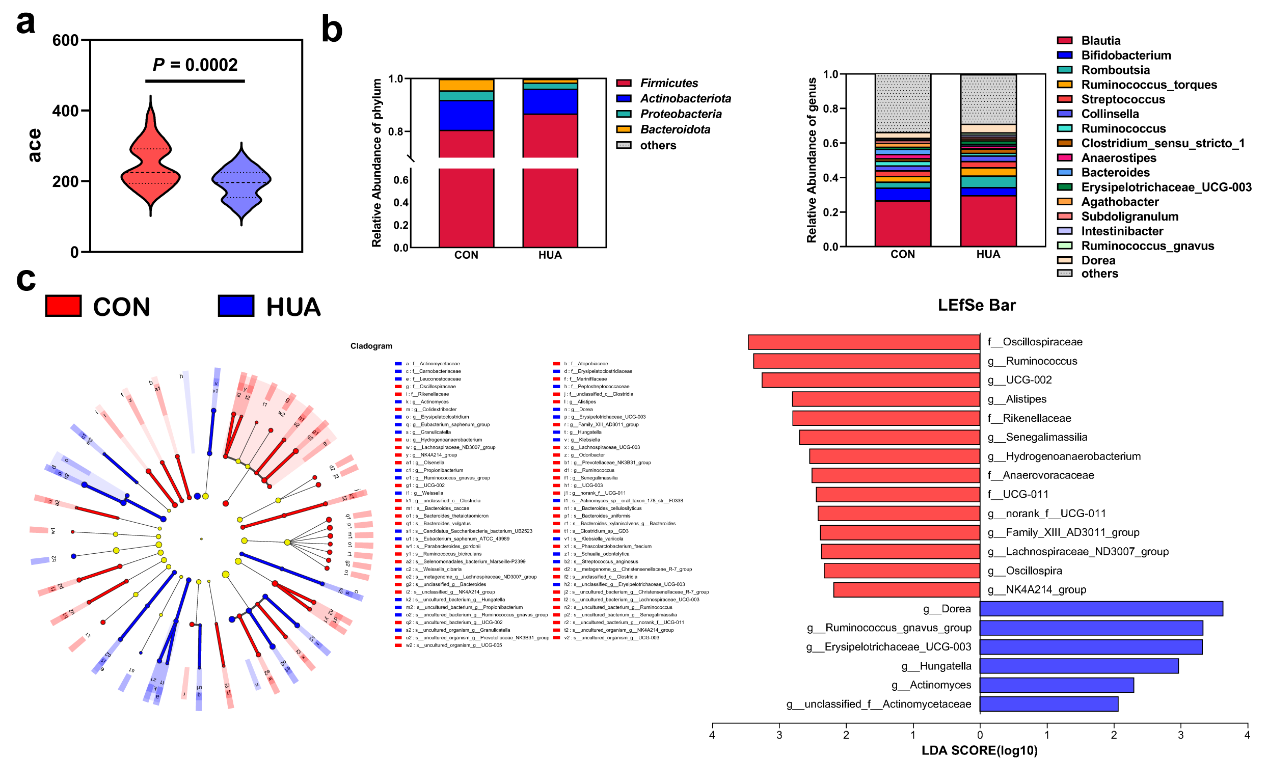
Supplementary Figure 9.** **Changes in gut microbiota abundance in HUA population. a**, Ace index of indicated groups based on alpha diversity analysis (CON, n = 32; HUA, n = 33). **b**, The alteration trends of the bacterial relative abundance in phylum and genus level (CON, n = 32; HUA, n = 33). **c**, LDA scores in Differential flora enrichment analysis between CON group (n = 32) and HUA group (n = 33). Data with error bars represent mean ± s.e.m. For a, data were analysed by two-tailed unpaired Student’s t test.

**
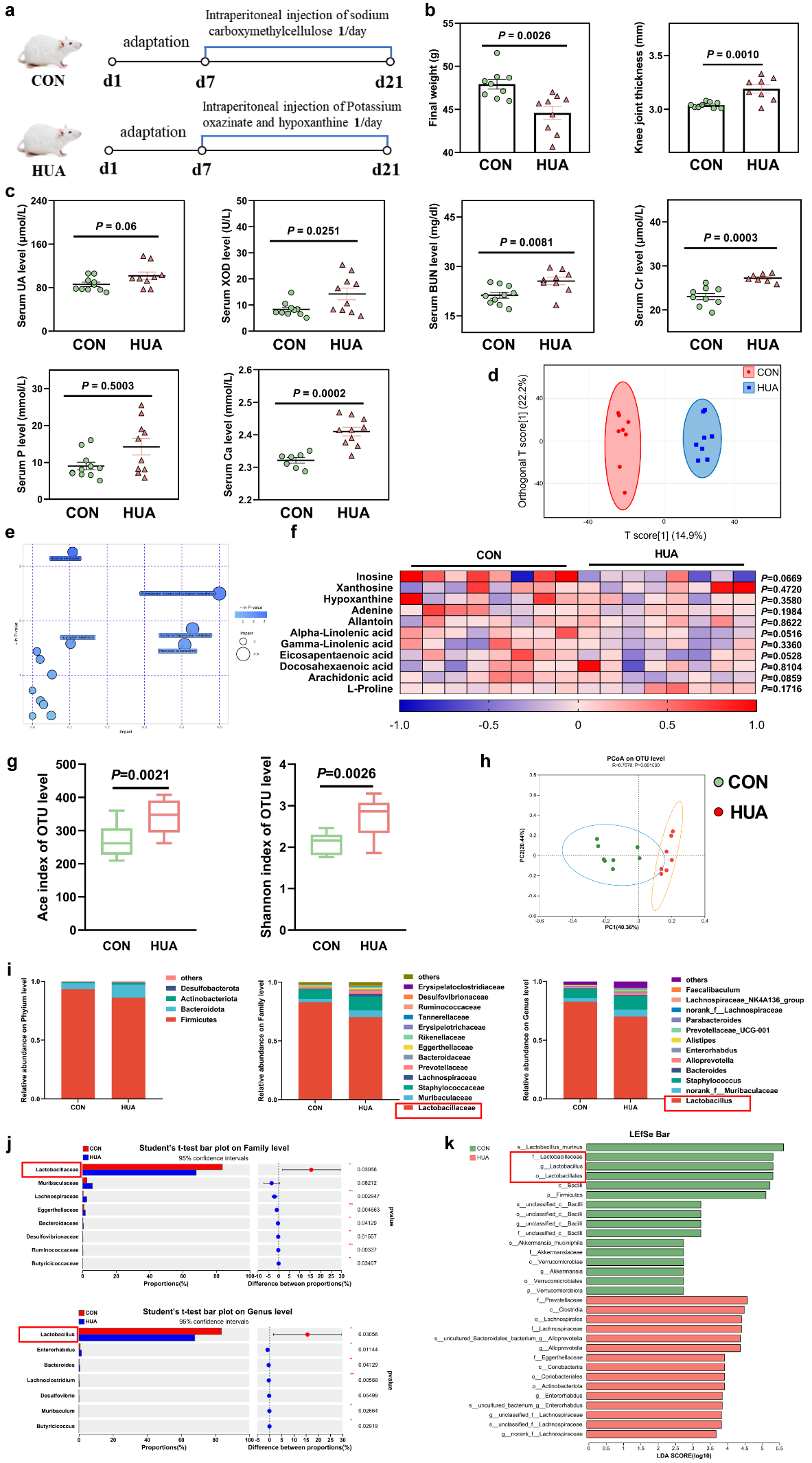
Supplementary Figure 10.** **The HUA model in mice did not achieve the desired effect. a**, Experimental design. Five-week-old (25 g) mice were divided into two groups: CON group (n=10) and HUA group (n=10). CON: control, sodium carboxymethylcellulose treatment; HUA: hyperuricemia, potassium oxyzincate and hypoxanthine treatment. **b**, Changes in final weight and knee joint thickness after HUA treatment (n = 10). **c**, Effects of HUA treatment on serum UA, XOD, BUN, Cr, P and Ca in mice (n = 10). **d**, Scores scatter plot of PCA model for group HUA vs CON, with serum samples all within the 95% confidence interval (Hotelling's T-squared ellipse). Squares indicate the HAU group (blue, n = 8) and circles indicate the CON group (red, n = 8). **e**, Pathway analysis for group HUA vs CON. Horizontal coordinates and bubble size indicate the magnitude of the influence factor of the pathway in the topological analysis, while vertical coordinates and bubble color indicate the p-value of the enrichment analysis (taking the negative natural logarithm, i.e. -ln(p). **f**, Changes in serum metabolites related to purine metabolism are shown on a heat map, with red indicating high expression of the substance in the group in which it is found and blue indicating low expression of the substance in the group in which it is found. The change in metabolites related to purine metabolism is shown as a heat map (n = 8). **g**, Ace and Shannon alpha diversity in the caecum faeces. Ace index HUA versus CON *P* = 0.0021; Shannon index HUA versus CON *P* = 0.0026. **h**, PCoA plots of weighted UniFrac distances based on OTUs from fecal samples of eight-week-old mice (R = 0.707, *P* = 0.001). The red color represents the HUA group (n = 7), while the green color represents the CON group (n = 8). **i**, Changes in the relative abundance of gut microbes at the phylum, family, and genus levels. **j**, student's t-test analyses on family and genus level. **k**, LDA scores in differential flora enrichment analysis between HUA group and CON group. Data with error bars represent mean ± s.e.m. For b, c, f, g and j, data were analysed by two-tailed unpaired Student’s t test.

**Supplementary Table 1. Antibiotics used in this study.**

| **Antibiotic** | **Source** | **Identifier** | |
| --- | --- | --- | --- |
| GLUT9 (1:1000) | Proteintech | 26486-1-AP |  |
| OAT1 (1:1000) | Abcam | ab135924 |  |
| URAT1 (1:1000) | Bioss | bs-10357R |  |
| ABCG2 (1:1000) | Abcam | ab108312 |  |
| PPAT (1:1000) | Proteintech | 15401-1-AP |  |
| PRRS (1:1000) | Bioss | bs-4504R |  |
| XOD (1:1000) | Abcam | ab109235 |  |
| ADA (1:1000) | Proteintech | 13328-1-AP |  |
| CNT2 (1:500) | Affinity Biosciences | DF4522 |  |
| TJP1 (1:2000) | Proteintech | 21773-1-AP |  |
| β-actin (1:5000) | Proteintech | 60009-1-Ig |  |

**Supplementary Table 2. Strains, plasmids, mutants, and primers used in this study.**

| **Strains** | | **Description** | **Source** | |  |  |
| --- | --- | --- | --- | --- | --- | --- |
| *Lactobacillus rhamnosus* | |  | Lab stock | |  |  |
| *E. coli GB2005* | | (HS996, ∆recET, ∆ybcC). The endogenous recET locus and the DLP12 prophage ybcC, which encodes a putative exonuclease similar to the  Redα, were deleted | Lab stock | |  |  |
| *E. coli GB05-dir* | | (GB2005, araC-BAD-ETgA) recE, recT, redγ and recA under BAD  promoter was inserted at the ybcC locus | Lab stock | |  |  |
| *Nissle1917/p15A-cm-Pgenta* | | The Pgenta promoter gene was heterologous expressed in E. coli Nissle1917 | This study | |  |  |
| *Nissle1917/p15A-cm-LGGiunH* | | The LGGiunH gene was heterologous expressed in E. coli Nissle1917 | This study | |  |  |
| *Nissle1917/p15A-cm-LGGpbuX* | | The LGGpbuX gene was heterologous expressed in E. coli Nissle1917 | This study | |  |  |
| *Nissle1917/p15A-cm-LGGdeoD* | | The LGGdeoD gene was heterologous expressed in E. coli Nissle1917 | This study | |  |  |
| *Nissle1917/p15A-cm-LGGABCT* | | The LGGABCT gene was heterologous expressed in E. coli Nissle1917 | This study | |  |  |
| **Plasmids** | | **Characteristics** | **Source** | |  |  |
| pBBR1-Rha-redγβα-kan | | pBBR1 replicon, kmR, redγβα under the control of Rha promoter | Lab stock | |  |  |
| p15A-cm-tetR-tetO-hyg-ccdB | | p15A replicon, c*mR* | Lab stock | |  |  |
| pMSP3535 | | pBR322 replicon, ermR, repE, nisR, nisK, under the control of T7 promoter | Lab stock | |  |  |
| RK2-pBAD-Cre-Dre-sacB | | pBAD replicon, apraR, kmR | Lab stock | |  |  |
| pNBU2-erm-genta-cspRecT | | pNBU2 replicon, gentaR | Lab stock | |  |  |
| pBBR1-Rha-redγβα-Kan-erm | | pBBR1 replicon, kmR, redγβα under the control of Rha promoter | Lab stock | |  |  |
| p15A-cm-Pgenta | | p15A replicon, cmR under the control of Pgenta promoter | This study | |  |  |
| p15A-cm-LGGiunH | | p15A replicon, cmR, LGGiunH under the control of Pgenta promoter | This study | |  |  |
| p15A-cm-LGGpbuX | | p15A replicon, cmR, LGGpbuX under the control of Pgenta promoter | This study | |  |  |
| p15A-cm-LGGdeoD | | p15A replicon, cmR, LGGdeoD under the control of Pgenta promoter | This study | |  |  |
| p15A-cm-LGGABCT | | p15A replicon, cmR, LGGABCT under the control of Pgenta promoter | This study | |  |  |
| p15A-cm-HA-erm-scaB-GpbuX | | p15A replicon, cmR, scaB under the control of erm promoter | This study | |  |  |
| p15A-cm-HA-erm-scaB-GiunH | | p15A replicon, cmR, scaB under the control of erm promoter | This study | |  |  |
| p15A-cm-HA-erm-scaB-GABCT | | P15A replicon, cmR, scaB under the control of erm promoter | This study | |  |  |
| **Mutants** | | **Characteristics** | **Source** | |  |  |
| LGG△iunH | | The deletion of 683 bp (873774—874456) region on L. rhamnosus chromosome was replaced by a linearized fragment of a plasmid of p15A-cm-HA-erm-scaB-GiunH | This study | |  |  |
| LGG△pbuX | | The deletion of 756 bp (1537889—1538646) region on L. rhamnosus chromosome was replaced by a linearized fragment of a plasmid of p15A-cm-HA-erm-scaB-GpbuX | This study | |  |  |
| LGG△ABCT | | The deletion of 833 bp (2376859—2377693) region on L. rhamnosus chromosome was replaced by a linearized fragment of a plasmid of p15A-cm-HA-erm-scaB-GABCT | This study | |  |  |
| **Primers** | | **Sequence** | | **Application** | | |
| p15A-5 | | AAACTACCGCATTAAAGCTT | | For amplification of p15A-cm supporter | | |
| p15A-3 | | CTGAACCGACGACCGGGTCG | |  |  |  |
| p15A-Pgenta-5 | | CAGAAATTCGAAAGCAAATTCGACCCGGTCGTCGGTTCAGGAAGGCACGAACCCAGTTG | | For amplification of Pgenta promotor | | |
| genta-60kDa-3 | | CGTTGCTGCTCCATAACAT | |  |  |  |
| Pgenta-LGiunH-5 | | TACGCCGTGGGTCGATGTTTGATGTTATGGAGCAGCAACGGAGGGCTTTAATCTATGAT | | For amplification of LGG-iunH gene | | |
| LGiunH-3 | | TTTGACAGCTTATCATCGATAAGCTTTAATGCGGTAGTTTCTAAAAATTAGCCGTTTTA | |  |  |  |
| Pgenta-LGpbuX-5 | | TACGCCGTGGGTCGATGTTTGATGTTATGGAGCAGCAACGCTAGGCTCGAAGATGAAGA | | For amplification of LGG-pbuX gene | | |
| LGpbuX-3 | | TTTGACAGCTTATCATCGATAAGCTTTAATGCGGTAGTTTTTATTTGTCTGAATCCTGT | |  |  |  |
| Pgenta-LGdeoD-5 | | TACGCCGTGGGTCGATGTTTGATGTTATGGAGCAGCAACGGAGGGCTTTAATCTATGAGTACACATATT | | For amplification of LGG-deoD gene | | |
| LGdeoD-3 | | TTTGACAGCTTATCATCGATAAGCTTTAATGCGGTAGTTTTTAACGTACAGGAATCTTCTTAGCGACGC | |  |  |  |
| Pgenta-LGABCT-5 | | TACGCCGTGGGTCGATGTTTGATGTTATGGAGCAGCAACGCTAGGCTCGAAGATGCAAACAATCATGCA | | For amplification of LGG-ABCT gene | | |
| LGABCT-3 | | ATTCTCATGTTTGACAGCTTATCATCGATAAGCTTTAATGCGGTAGTTTTTATTCACCC | |  |  |  |
| erm-5 | | GTTCTATGCTTTCTTTTTGTAGCCGGCTAAACGGATAGTCCCCCAAAATCATCTTGCCTTTGATATTGAGGTATCATTT | | For amplification of erm resistance gene | | |
| erm-3 | | CCTTTTTAATCACAATTCAGAAAATATCATAATATCTCATTTCACTAAATAATAGTGAACTTAGCCGTTAAATATTTTA | |  |  |  |
| sacB-5 | | GTTCACTATTATTTAGTGAA | | For amplification of sacB gene | | |
| sacB-3 | | AGTGTGACTCTAGTAGAGAGCGTTCACCGACAAACAACAGTTTGTTAACTGTTAATTGT | |  |  |  |
| P15A-GPHAR-3 | | GTTGCTGGCAACCCCGGCGGATGTCGATCGTGCCAAACAATTGCTGCAGCGACGTCGATATCTGGCGAA | | For Construction of p15A-cm-HA-erm-scaB-GpbuX plasmids | | |
| GP-HAL-5 | | GTAAAGTGCAACGATGCCCC | |  |  |  |
| GP-HAL-3 | | CAATTGTTGAGCAAAAATGTCTTTTACGATAAAATGATACCTCAATATCATCGCCTCGGCTTTGCAGAC | |  |  |  |
| GP-HAR-5 | | GCTGCAGCAATTGTTTGGCA | |  |  |  |
| GP-HAR-3 | | TCGGGTTCAGTGTTGGTGCCGATTTTGATTGGGGCATCGTTGCACTTTACCACAACTTTTTCGCAAAAT | |  |  |  |
| Gi-HAL-5 | | GACATTCCCGGCAATCGGAC | | For Construction of p15A-cm-HA-erm-scaB-GiunH plasmids | | |
| Gi-HAL-3 | | AAGGCAAGATGATTTTGGGG | |  |  |  |
| Gi-HAR-5 | | TTTGAACACTCATGTTTAAC | |  |  |  |
| Gi-HAR-3 | | GATGGATGTTCAACTAATTTGTCCGATTGCCGGGAATGTCGCTGATTGATCTGAAAGGA | |  |  |  |
| p15a-sacB-3 | | TGTTAGTCATTTTCTTTCTCAACCTCGTCATTGGCACCAAGTTAAACATGAGTGTTCAAAGACGTCGATATCTGGCGAA | |  |  |  |
| GA-HAL-5 | | CAAGTGTTGCGACAGACTGC | | For Construction of p16A-cm-HA-erm-scaB-GABCG plasmids | | |
| GA-HAL-3 | | CAATTGTTGAGCAAAAATGTCTTTTACGATAAAATGATACCTCAATATCATCTTTTGGCTGCAACCAGA | |  |  |  |
| GA-HAR-5 | | ACGCACAGCATGTGAATATT | |  |  |  |
| GA-HAR-3 | | TGCGTGATCATCAAAATGCCGTGGGTGTGGGCAGTCTGTCGCAACACTTGTGGCCTGATGCCGCTGCCG | |  |  |  |
| P15A-GAHAR-3 | | CATTTTAAAAAAATCTACGGTTAGTTTATAAATATTCACATGCTGTGCGTGACGTCGATATCTGGCGAA | |  |  |  |
| check-LGiunH-5 | | CCAATCCAGCAGTAAAATGA | | For the first step of single-exchange recombinant colony PCR detection | | |
| check-LGiunH-3 | | AATTCCGGAACCGCTCATAT | |  |  |  |
| check-LGABCT-5 | | TGTGTTGGCGGCCTTTAAGA | |  |  |  |
| check-LGABCT-3 | | GGAGCCTAATCTGACCAGAT | |  |  |  |
| check-LGpbux-5 | | TACAATTTTGGCCCCGGGAA | |  |  |  |
| check-LGpbux-3 | | CGGTTGTGTTGGGGTGTGCA | |  |  |  |
| **Primers** | | **Sequence** | | **Gene number** | | |
| 16S-5: | | AGAGTTTGATCCTGGCTCAG | |  |  |  |
| 16S-3: | | GGTTACCTTGTTACGACTT | |  |  |  |
| proW-5 | | TGATTCCTTCGTGGACTGGC | | WP_005684724_1 |  |  |
| proW-3 | | TCAGAACCAAGGTCAGCGTC | |  |  |  |
| dnaE-5 | | GCGCAAAGTGGGGTTACTTG | | WP_014569626_1 |  |  |
| dnaE-3 | | GCAAGCGTTGGTGCTTTGTA | |  |  |  |
| proV-5 | | CACTGCGAATGCTGAATCGG | | WP_005713097_1 |  |  |
| proV-3 | | GGCAAACCCGTCTAATCCCA | |  |  |  |
| iunH-5 | | ACTAGGTGCCACTGACGTAC | | WP_106011914_1 |  |  |
| iunH-3 | | GGTTGGCACTAGGGTCAGAT | |  |  |  |
| pbuX-5 | | TTCGGGATCGTTGGCATTCA | | WP_005688928_1 |  |  |
| pbuX-3 | | ACTGCGGCTACACTACCAAC | |  |  |  |
| deoD-5 | | TTTCAGCTTTTGCGTGAGGC | | WP_014569004_1 |  |  |
| deoD-3 | | TAACGCCTGTGCATGGAACT | |  |  |  |
| ABCT-5 | | GGTTTGGCTTGCTTCCACTG | | WP_005685974_1 |  |  |
| ABCT-3 | | TCAGAGAGGTCCGTTGTTGC | |  |  |  |
| β-actin-5 | | GCACCCAGCACAATGAAGAT | | NM_001101.5 |  |  |
| β-actin-3 | | CATCTGCTGGAAGGTGGACA | |  |  |  |
| PRPS-5 | | GCCGTGATCGCTTAGTGGAG | | NM_001204402.2 |  |  |
| PRPS-3 | | GCCACAACCACTCTGAACAATG | |  |  |  |
| PPAT-5 | | TCACACAAGGGAATGGGTCTT | | NM_002703.5 |  |  |
| PPAT-3 | | TCAACAACGAAGGGCTGACA | |  |  |  |
| ADA-5 | | TCTGGGACATGGAGCTCAGA | | NM_000022.4 |  |  |
| ADA-3 | | TGCTATCAGTTTGGCTGGGC | |  |  |  |
| XOD-5 | | GGCCAGATACAAGGTTGGCT | | NM_000379.4 |  |  |
| XOD-3 | | GTGTTGGAGGGAAGGTTGGT | |  |  |  |
| PRODH-5 | | GCTGGAATTTGTGATGAGAGAGT | | NM_001195226.2 |  |  |
| PRODH-3 | | TCCTCCCCGGCTACAAAATG | |  |  |  |
| HGPRT-5 | | CCTGGCGTCGTGATTAGTGA | | NM_000194.3 |  |  |
| HGPRT-3 | | CGAGCAAGACGTTCAGTCCT | |  |  |  |
| ADSS2-5 | | TGGTTGGACCTCGTTTTGCT | | NM_001126.5 | | |
| ADSS2-3 | | AGACTTCTTGGTTTGCTGGGA | |  |  |  |
| β-actin-5 | | AATCCTGCGGCATCCACGAAAC | | XM_021086047.1 | | |
| β-actin-3 | | CTCCTGCTTGCTGATCCACATCTG | |  |  |  |
| SLC28A2/CNT2-5 | | AGCCCTTGGCTTTCCGTTAT | | XM_013993042.2 | | |
| SLC28A2/CNT2-3 | | TGGCAACTAGAAGCAAGGTTTG | |  |  |  |
| ABCG2-5 | | ATAAGAGTTCCTGAGATTGGAGCC | | NM_214010.1 | | |
| ABCG2-3 | | GGTGTTCCTTTTTGACATCGGG | |  |  |  |
| SLC2A9/GLUT9-5 | | GGTACGGACGTGCCATAGAC | | XM_021101011.1 | | |
| SLC2A9/GLUT9-3 | | ACACCCATGAGGAAACGTCC | |  |  |  |
| TJP1-5 | | TCAAGGTCTGCCGAGACAAC | | XM_021098856.1 | | |
| TJP1-3 | | ATCACAGTGTGGTAAGCGCA | |  |  |  |
| β-actin-5 | | TCCTGCGGCATCCACGAGA | | NM_007393.5 | | |
| β-actin-3 | | CCGCCGATCCAGACCGAGTA | |  |  |  |
| GLUT9-5 | | TGGCAGGTCATTACTGTGGTTGTC | | NM_001012363.2 | | |
| GLUT9-3 | | CGTCCGAGCCGCTCAATAACTAAG | |  |  |  |
| URAT1-5 | | GGCTTCACCTTCTACGGCCT | | NM_009203.3 | | |
| URAT1-3 | | AGCAGCAGGGTGCCGATCTT | |  |  |  |
| OAT1-5 | | CTGCATCTTCCTGTACACTG | | NM_008766.3 | | |
| OAT1-3 | | CGTAGATGAAGAGAGGCATG | |  |  |  |
| ABCG2-5 | | CCTGAGATGTTAGAGTCCCC | | NM_001355477.2 | | |
| ABCG2-3 | | CAGTCTAGTGAGGCTACTGG | |  |  |  |
| β-actin-5 | | GATGACGATATTGCTGCGCTC | | NM_0013101421.1 | | |
| β-actin-3 | | TCGATGGGGTACTTGAGGGT | |  |  |  |
| CNT2-5 | | GAAGGTCTTGGCTCAACCGA | | NM_004212.4 | | |
| CNT2-3 | | CTTGTCGTTGCATGTTGGCA | |  |  |  |
| TJP1-5 | | CTCCCTACTCACCACAAGCG | | XM_013104936.1 | | |
| TJP1-3 | | CCTATACACCTGTTGAGAGGCA | |  |  |  |
| ABCG2-5 | | AAAGTGGCCGATTCCAAGGT | | XM_048074518.1 | | |
| ABCG2-3 | | GGCCAACAAGGTGAGGCTAT | |  |  |  |
| GLUT9-5 | | TGGCAGGTCATTACTGTGGTTGTC | | XM_013099415.3 | | |
| GLUT9-3 | | CGTCCGAGCCGCTCAATAACTAAG | |  |  |  |

Figure 2


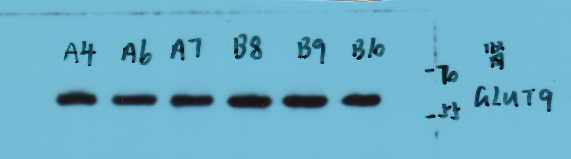

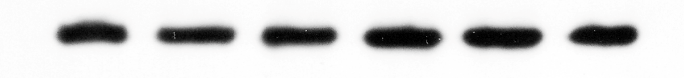

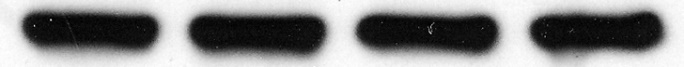

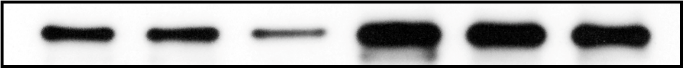

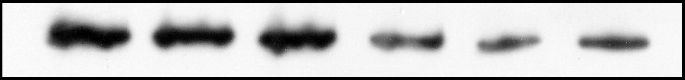

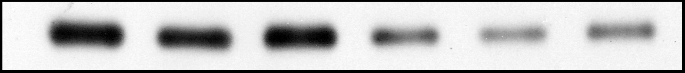

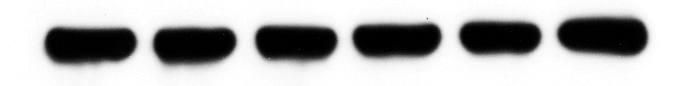

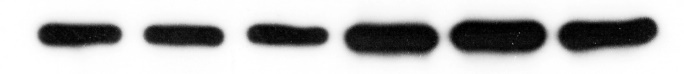

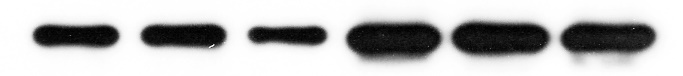

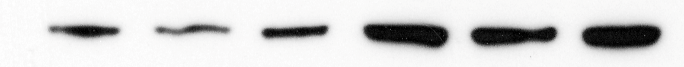

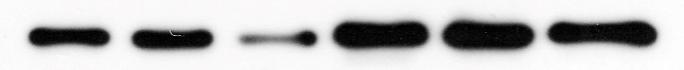

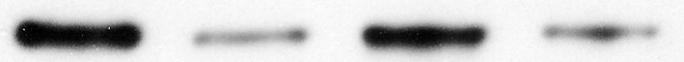

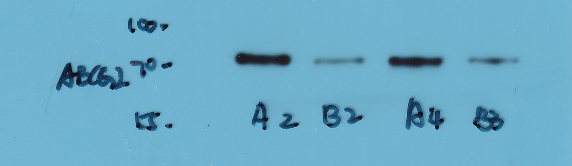

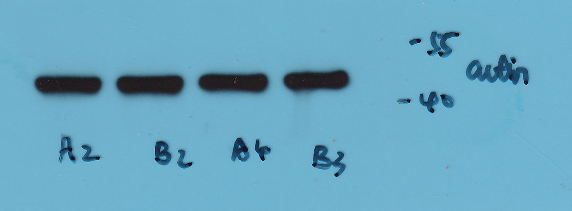

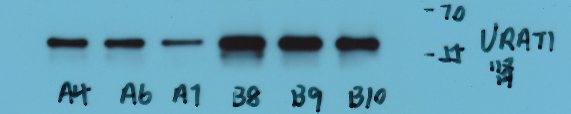

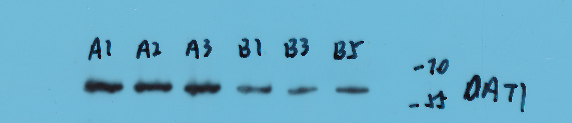

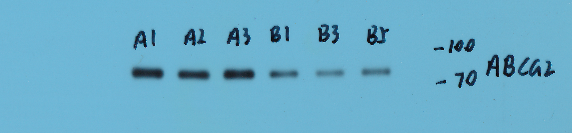

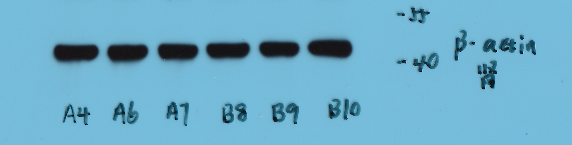

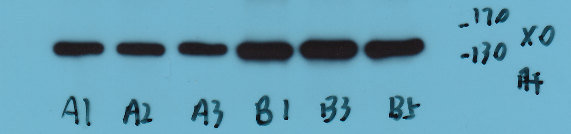

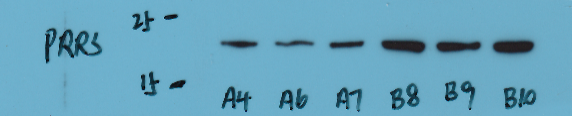

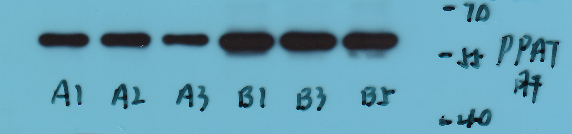

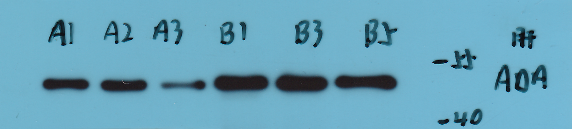

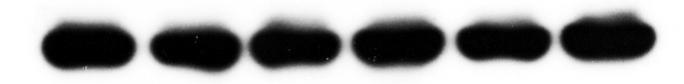

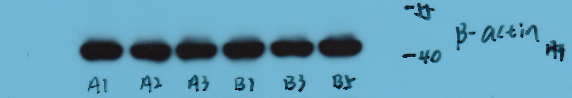


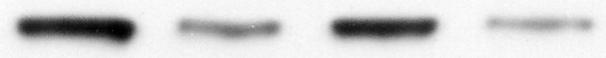

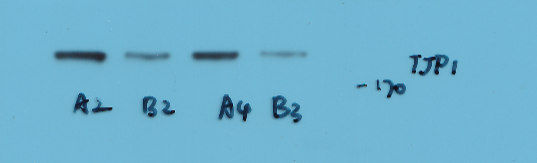


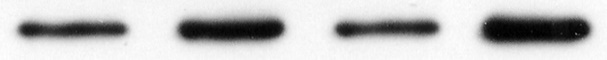

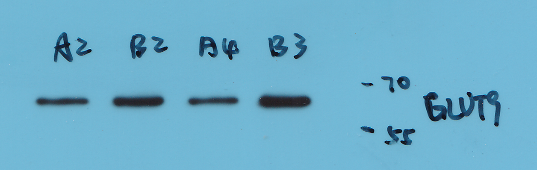

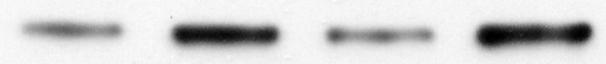

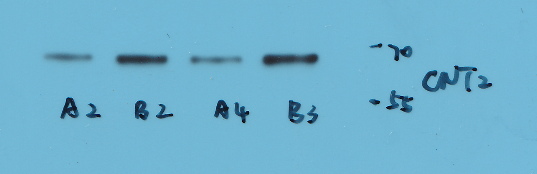


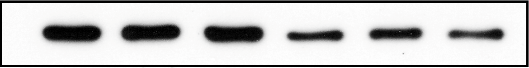

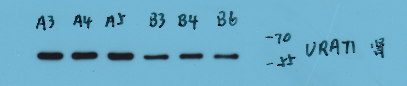

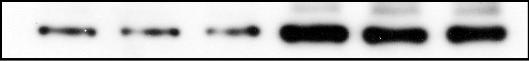

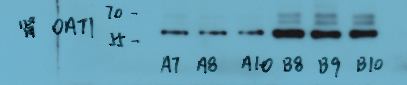

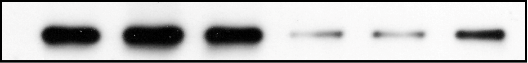

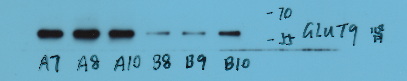

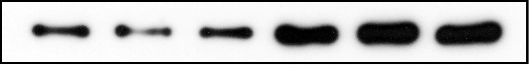

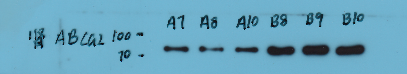

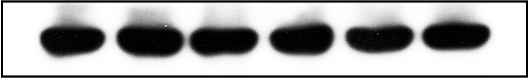

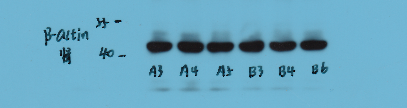

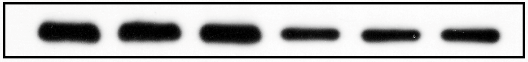

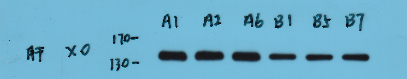

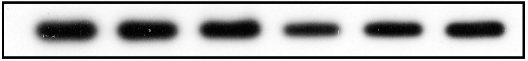

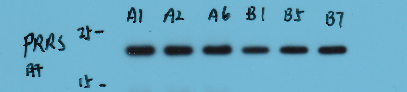

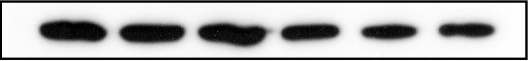

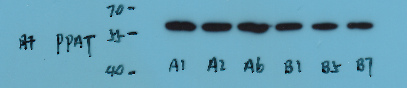

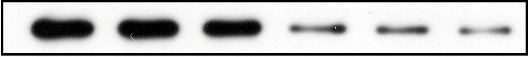

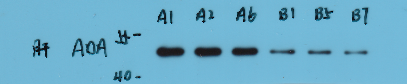

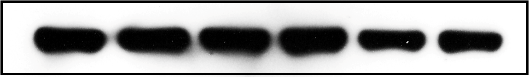

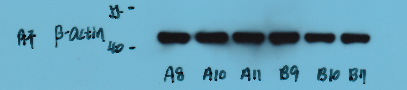
Figure 3

Figure 4


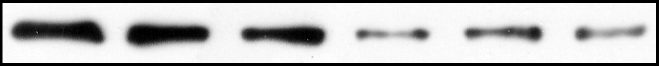

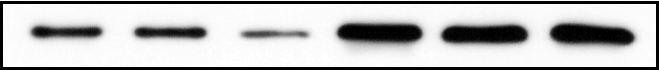

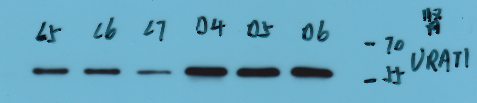

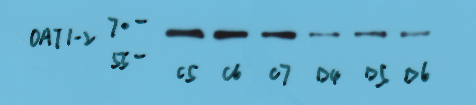

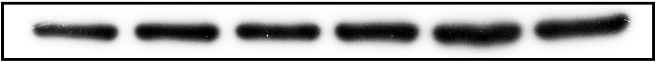

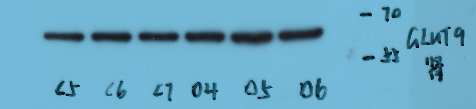

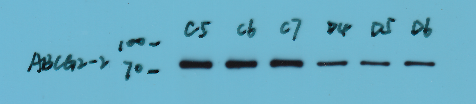

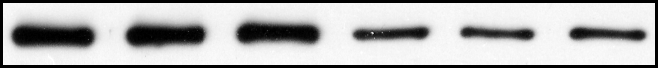

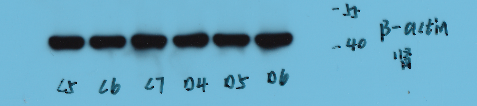

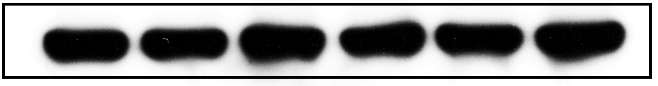


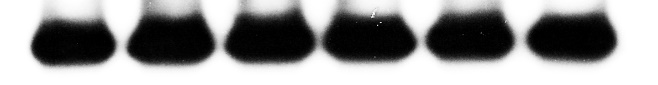

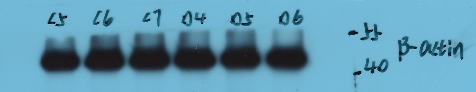


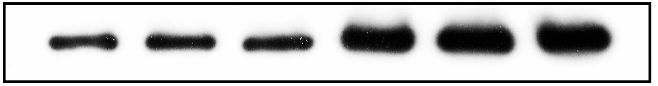

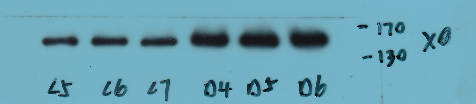

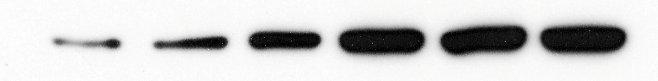

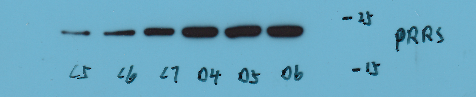

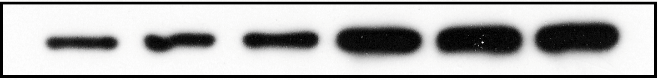

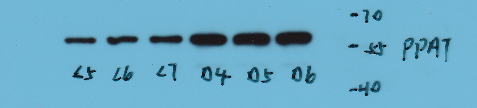

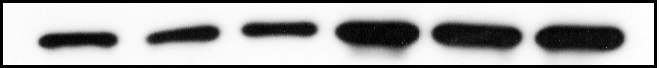

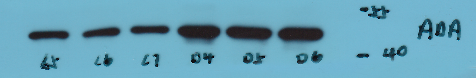


Figure 8


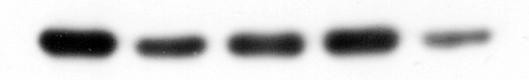

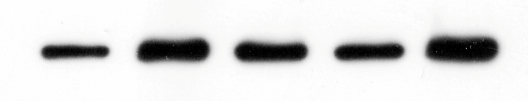

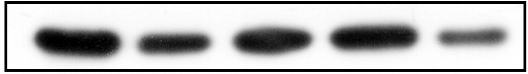

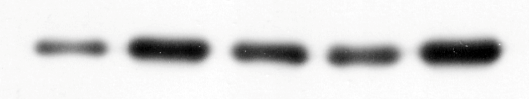

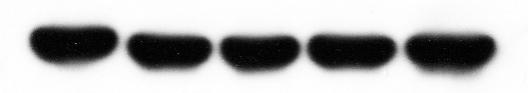

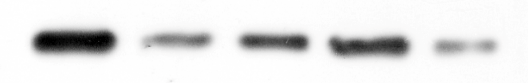

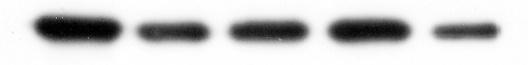

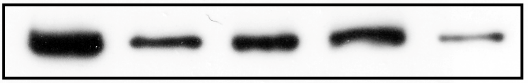

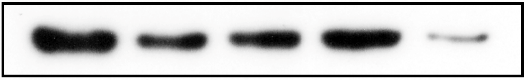

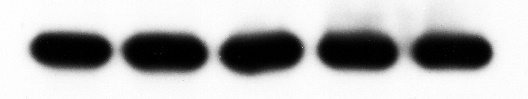

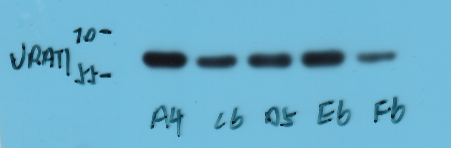

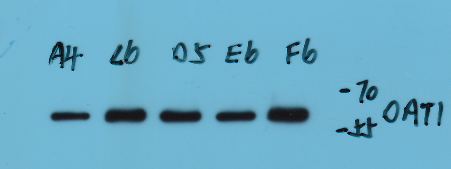

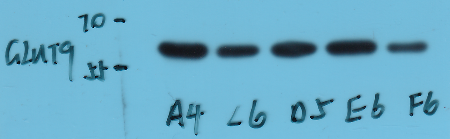

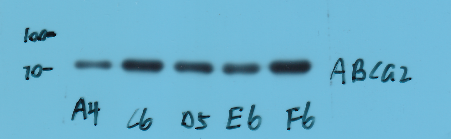

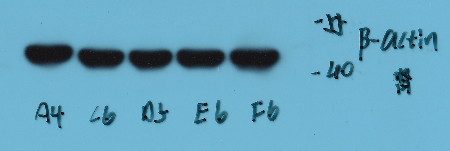

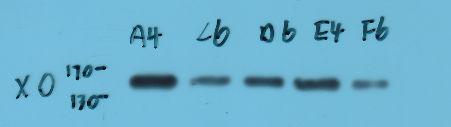

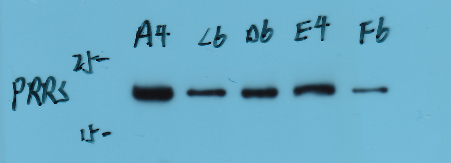

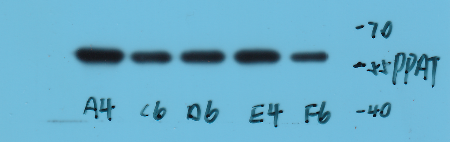

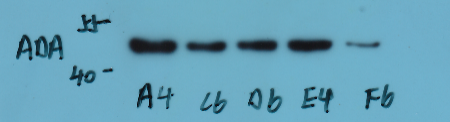

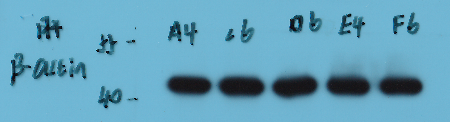

Supplement: Supplementary file 1 — Supplementary Information [file 41522_2024_486_MOESM1_ESM.docx]
